# Evaluating Effects of Multilevel Interventions on Disparity in Health and Healthcare Decisions

**DOI:** 10.1007/s11121-024-01677-8

**Published:** 2024-06-22

**Authors:** John W. Jackson, Yea-Jen Hsu, Lauren C. Zalla, Kathryn A. Carson, Jill A. Marsteller, Lisa A. Cooper, the RICH LIFE Project Investigators

**Affiliations:** 1 Department of Epidemiology, Johns Hopkins Bloomberg School of Public Health, Baltimore, MD USA; 2 Department of Biostatistics, Johns Hopkins Bloomberg School of Public Health, Baltimore, MD USA; 3 Department of Mental Health, Johns Hopkins Bloomberg School of Public Health, Baltimore, MD USA; 4 Johns Hopkins Center for Health Equity, Johns Hopkins University, Baltimore, MD USA; 5 Department of Health Policy & Management, Johns Hopkins Bloomberg School of Public Health, Baltimore, MD USA; 6 Department of Medicine, Johns Hopkins School of Medicine, Baltimore, MD USA; 7 Welch Center for Prevention, Epidemiology, & Clinical Research, Baltimore, MD USA; 8 Department of Health Behavior & Society, Johns Hopkins Bloomberg School of Public Health, Baltimore, MD USA

**Keywords:** Multilevel, Intervention, Evaluation, Disparity, Equity, Decision, Causal inference, Allowability, Trial

## Abstract

In this paper, we introduce an analytic approach for assessing effects of multilevel interventions on disparity in health outcomes and health-related decision outcomes (i.e., a treatment decision made by a healthcare provider). We outline common challenges that are encountered in interventional health disparity research, including issues of effect scale and interpretation, choice of covariates for adjustment and its impact on effect magnitude, and the methodological challenges involved with studying decision-based outcomes. To address these challenges, we introduce total effects of interventions on disparity for the entire sample and the treated sample, and corresponding direct effects that are relevant for decision-based outcomes. We provide weighting and g-computation estimators in the presence of study attrition and sketch a simulation-based procedure for sample size determinations based on precision (e.g., confidence interval width). We validate our proposed methods through a brief simulation study and apply our approach to evaluate the RICH LIFE intervention, a multilevel healthcare intervention designed to reduce racial and ethnic disparities in hypertension control.

**Supplementary Information:**

The online version contains supplementary material available at 10.1007/s11121-024-01677-8.

## Introduction

Often the effect of an intervention on disparity is evaluated through the lens of effect (measure) modification where the effect is compared across levels of the social group (e.g., race) on the additive or relative scale. There are a few problems with this approach. First, due to differences in the outcome among the referent social group in the treatment and control arms, the intervention effect on disparity can differ depending on whether disparity is measured as a difference or a ratio. Commonly used regression estimators typically emphasize one scale (e.g., ratio for binary measures) rather than how investigators conceive of what constitutes change in disparity. Second, a meaningful measure of disparity (e.g., in the outcome of hypertension control) may adjust for certain allowable covariates (e.g., age and gender) but avoid adjusting for non-allowable covariates (implicated in generating disparity, e.g., pre-existing conditions and socioeconomic status) (Cook et al., 2009; Duan et al., 2008; Jackson, 2021). Regression estimators that are often used to gain precision or address potential bias typically do so by conditioning on all covariates which may overadjust (and possibly underestimate) the disparity. Third, in the setting of healthcare, it will often be important to assess effects on process outcomes such as the disparity in treatment decisions by clinicians. The measure of disparity in treatment decision-making should account for relevant criteria (e.g., clinical needs at the time of the decision, such as systolic blood pressure for antihypertensive treatment decisions). But these criteria may have in fact been affected by the intervention and adjusting for such factors in a regression model can introduce bias. This dilemma makes it difficult to study the effects of multilevel healthcare interventions on disparity in treatment decision-making.

We will present an analytic approach for evaluating intervention effects on disparity in health and healthcare decisions (i.e., treatment decisions made by a healthcare provider). Based on the potential outcome framework for causal inference (Robins, 1986; Rubin, 1974), this approach allows researchers to choose the effect scale and the covariate adjustment set that match their equity value judgments while resolving confounding by other measured covariates that are imbalanced across intervention arms. When certain process outcomes are of interest (e.g., treatment decisions), the approach provides a novel direct effect that captures effects on disparity in the process outcome that appropriately accounts for allowable covariates that may be affected by the intervention, but without over-adjusting the disparity measure for the process outcome.

The paper is organized as follows. We begin with our motivating example which concerns a cluster-randomized trial of a multilevel intervention to reduce disparities in hypertension control. We then review the issues outlined above, describe our analytic approach, and apply our methods to our motivating example. We close by noting limitations, implications for study design, and distinctions from other approaches in the literature. As our intended audience is primarily applied, formal results and proofs appear in the supplement. Procedures for sample size determination based on precision are also provided in the Supplemental Material.

## Motivating Example: Hypertension Control

Despite the availability of effective therapy and prevention strategies, racially and ethnically minoritized groups in the USA remain disproportionately burdened by cardiovascular disease—a leading cause of death—in large part due to uncontrolled hypertension (Murphy et al., 2013). Barriers to hypertension control are multifactorial and operate at multiple levels of societal organization, including the individual patient, familial and support systems, clinical team, institutional, and municipal and policy levels (Mueller et al., 2015). Multilevel strategies to reduce disparities in hypertension control include patient activation, practice-based quality improvement efforts, such as audit and feedback interventions, and reorganization of clinical care teams (Hysong, 2009; Mills et al., 2018; Viswanathan et al., 2010; Walsh et al. 2005). Few multilevel interventions have been designed specifically to reduce disparities in hypertension control.

## Proposed Design

### Overview

The RICH LIFE Project (Cooper et al., 2020) was a multilevel, pragmatic, two-arm cluster-randomized trial to compare the effectiveness of two approaches for reducing disparities. Its goal was to test practical, scalable approaches to addressing disparities in hypertension control. It was designed using the Pragmatic-Explanatory Continuum Criteria Indicators (PRECIS) criteria for pragmatic trials, and through engagement with health system administrators, clinical staff, and community partners. The full protocol is described elsewhere (Cooper et al., 2020).

### Population

The trial enrolled 1820 adults (non-Hispanic Black [57%], non-Hispanic White [33%], Hispanic [10%]) during 2016–2019 across 30 primary care practices within five healthcare systems in Maryland and Pennsylvania. Adults were eligible if, by the time of eligibility assessment, they were 21 years of age or older, received care in the prior 6 months, were diagnosed with hypertension, had an uncontrolled systolic blood pressure ($\ge$ 140 mm Hg) at their recent office-based visit, and had at least one of the following cardiovascular risk factors: diabetes mellitus, hyperlipidemia, coronary heart disease, current tobacco smoking, or depression. Patients with pregnancy, certain serious medical conditions, substance use disorders, those no longer receiving care at the practice site, or those who declined consent were excluded.

### Intervention Arms

Within each participating healthcare system, clinical practice sites were randomized to receive the control condition “Standard of Care Plus” (SCP) or the treatment condition “Collaborative Care/Stepped Care” (CC/SC) for 1-year post randomization. Practice sites in the SCP arm received blood pressure measurement standardization with electronic monitors, blood pressure dashboards for audit-feedback, training modules on hypertension care best practices for providers and staff, and presentations on equity for system leaders. Practice sites in the CC/SC arm combined the SCP components with an intensive care management and stepped care model. The patient’s clinical care team was redesigned to include, at minimum, a primary care physician and a care manager (a registered nurse or licensed clinical social worker) whose role was to co-develop (with the patient) a medical management plan and to facilitate care coordination. Around 3-month follow-up, patients and their care managers co-determined whether the care team would be broadened to include either: (1) a community health worker to help patients assess and overcome barriers to self-management and effective interaction with providers, and/or (2) a consultation with a relevant clinical specialist (e.g., a cardiologist) to review the patient’s case and provide recommendations. While these collaborative and stepped care components affected processes at the clinical care team-level, they were only applied to enrolled patients seen at practice sites randomized to the CC/SC arm.

### Follow-Up and Outcome Ascertainment

Eligibility was assessed up to 3 to 6 months after the implementation of the blood pressure standardization, audit and feedback, and system-level interventions were implemented. Follow-up began after the point of enrollment and lasted for up to 2 years. Data on demographics, clinical status, experiences of care, and social determinants of health were collected through surveys at baseline, 12 months, and 24 months, supplemented by clinical data pulls from the electronic medical record. The primary clinical outcome was blood pressure control (< 140/90 mm Hg) at 12 months (via the closest office-based measurement in the electronic medical record between 6- and 18-month follow-up).

Text Box 1 Three common challenges arise when evaluating intervention effects on disparity: (1) effects on disparity can depend on whether disparity is defined in absolute or relative terms; (2) covariate adjustment via regression methods to address confounding, loss to follow-up, or to increase precision may inadvertently overadjust a disparity measure and impact the magnitude of intervention effects on disparity; (3) assessing intervention effects on disparate decision-making will often require accounting for relevant criteria that is possibly affected by the intervention, but regression methods cannot properly account for such criteria without introducing bias

## Common Challenges in Analysis and Interpretation

To further motivate our proposal, we discuss three common challenges that arise when analyzing and interpreting the impact of interventions on disparity. Let $\mu (z,r)$ represent $E[Y|Z=z,R=r]$ the mean of the outcome $Y$ measured at follow-up, e.g., controlled hypertension (1 = yes, 0 = no), for a given intervention arm $Z$ (1 = treatment, 0 = control) and a specific social group $R$ (1 = marginalized, e.g., Black, 0 = privileged, e.g., White). For example, $\mu (\mathrm{1,1})$ would represent $E[Y|Z=1,R=1]$ the average level of controlled hypertension at follow-up among the Black population in the treated arm. Because $Y$ is binary, this is equivalent to the proportion of controlled hypertension at follow-up in this group, $P(Y=1|Z=1,R=1)$. We may consider a conditional average given covariates ${\varvec{X}}$, $E[Y|Z=1,R=1,{\varvec{X}}={\varvec{x}}]$, as $\mu \left(z,r,{\varvec{x}}\right)$.

### Outcome Scale and Coding

Informally, we can express the intervention’s effect on disparity, expressed as $\tau$, by contrasting disparity among the treatment arm with disparity in the control arm. On the risk difference (i.e., additive) scale,

1 $${\tau }^{RD}=\theta \left(1\right)-\theta \left(0\right)$$

where $$\theta \left(z\right)=\left(\mu \left(Z=z,R=1)-\mu (Z=z,R=0\right)\right)$$

On the risk ratio or prevalence ratio (i.e., relative) scale,

2 $${\tau }^{RR}=\phi (1)/\phi (0)$$

where $$\phi \left(z\right)=\frac{\mu \left(Z=z,R=1\right)}{\mu \left(Z=z,R=0\right)}=1+\frac{\left(\mu \left(Z=z,R=1\right)-\mu (Z=z,R=0)\right)}{\mu \left(Z=z,R=0\right)}$$

From the perspective of Eqs. (1) and (2), the scale one uses to conceive of an effect (i.e., the difference in disparity from control versus treatment arms) is paramount in describing progress toward equity. The additive measure ${\tau }^{RD}$ emphasizes how the absolute disparity in the proportion of controlled hypertension differs between the treatment and control arms. In contrast, the prevalence or risk ratio measure ${\tau }^{RR}$ emphasizes how the absolute disparity compares to the level of controlled hypertension among White participants, and how this ratio differs across treatment and control arms. An odds ratio ${\tau }^{OR}$ (not shown) behaves similarly, emphasizing how the absolute disparity in the odds compares to the odds of controlled hypertension among White participants, and how this ratio differs across treatment and control arms.

Concerningly, intervention effects on the additive and relative scales can be in conflict, with one indicating reduced disparity, and the other indicating no change or increased disparity. Supplemental Table 1 replicates a hypothetical scenario (Asada, 2010) where either ${\tau }^{RD}$ or ${\tau }^{RR}$ is null and the other is not. There are striking empirical examples (Harper et al., 2010) where disparity (across time rather than across intervention arms) decreases on the difference scale but increases on the ratio scale. Exclusive use of ${\tau }^{RD}$ or ${\tau }^{RR}$ may prioritize one form of change (i.e., additive or ratio) in characterizing the intervention effect. When, for binary outcomes, one reports ${\tau }^{RR}$, another issue arises. The degree of change can also depend on whether the outcome is coded as an attainment (e.g., controlled hypertension) or a shortfall (e.g., uncontrolled hypertension). For example, Supplemental Table 1 shows a hypothetical scenario (Kjellsson et al., 2015) where ${\tau }^{RR}$ can be null when outcomes are coded as attainments but non-null when otherwise coded as shortfalls. Disparity may be reported on both scales to provide a fuller picture (Harper et al., 2010; Kjellsson et al., 2015).^1^

**Table 1:** Average potential outcomes and total effect estimates (SATE-D) under the treatment versus control conditions of the RICH LIFE intervention on uncontrolled and controlled hypertension outcomes at 2 years’ follow-up by analytical approach,

	Weighting	G-computation
Uncontrolled hypertension		
Black mean (*R* = 1)		
Treatment (*Z* = 1)	0.43 (0.37, 0.50)	0.43 (0.37, 0.49)
Control (*Z* = 0)	0.46 (0.40, 0.53)	0.46 (0.40, 0.53)
White mean (*R* = 0)		
Treatment (*Z* = 1)	0.26 (0.15, 0.33)	0.25 (0.16, 0.33)
Control (*Z* = 0)	0.29 (0.23, 0.36)	0.29 (0.24, 0.35)
Intervention effect on disparity		
Additive (${\widetilde{\tau }}^{RD})$	0.01 (− 0.10, 0.14)	0.01 (− 0.11, 0.13)
Relative (${\widetilde{\tau }}^{RR})$	1.09 (0.75, 1.81)	1.09 (0.75, 1.75)
Controlled hypertension		
Black mean (*R* = 1)		
Treatment (*Z* = 1)	0.57 (0.50, 0.63)	0.57 (0.51, 0.63)
Control (*Z* = 0)	0.54 (0.47, 0.60)	0.54 (0.47, 0.60)
White mean (*R* = 0)		
Treatment (*Z* = 1)	0.74 (0.67, 0.85)	0.75 (0.67, 0.83)
Control (*Z* = 0)	0.71 (0.65, 0.76)	0.71 (0.65, 0.76)
Intervention effect on disparity		
Additive (${\widetilde{\tau }}^{RD})$	− 0.01 (− 0.14, 0.10)	− 0.01 (− 0.13, 0.10)
Relative (${\widetilde{\tau }}^{RR})$	0.99 (0.82, 1.19)	0.99 (0.83, 1.19)

Unfortunately, when applied researchers analyze intervention effects, the scale choice is driven less by their values about what sort of change represents an effect, but rather by statistical considerations. For example, regression models are used to adjust for covariates ${\varvec{X}}$ (for precision or to reduce bias). For binary outcomes, logistic regression (which provides ${\tau }^{OR}$) or a modified Poisson or negative binomial regression (which provide ${\tau }^{RR})$ are often used. However, a linear regression model for binary outcomes (which provides ${\tau }^{RD})$ can be difficult to estimate when adjusting for ${\varvec{X}}$ because the model can produce implausible predicted values. Thus, for binary outcomes, ${\tau }^{RD}$ is seldom reported which prioritizes relative rather than absolute effects (an implicit value judgment) when assessing the impact of an intervention on disparity. A modeling approach that allows adjustment for ${\varvec{X}}$ while reporting effects on both scales is desirable.

### Allowability

As before, we may informally express the intervention effect $\tau$ by contrasting the disparity in the control arm with the disparity in the treatment arm. To make our point explicit in this subsection we will consider effects on disparity within levels of covariates ${\varvec{X}}$. For example, consider the conditional additive effect:

3 $${\tau }^{RD}\left(x\right)=\psi \left(1,x\right)-\psi (0,x)$$

where $$\psi \left(z,x\right)=\mu \left(Z=z,R=1,x\right)-\mu (Z=z,R=0,x)$$

From the perspective of Eq. (3), the choice of what covariates are included in ${\varvec{X}}$ (if any) is paramount because it helps define what we mean by disparity (Jackson, 2021; Jackson et al., 2022). Our goal for the intervention, after all, is to minimize the treatment arm’s racial difference in outcomes, i.e., $\psi \left(1,{\varvec{x}}\right)$. We would include ${\varvec{X}}$ if we believe that racial differences among those similarly situated on ${\varvec{X}}$ better reflects what we mean by equity in hypertension control. For example, Black participants are often younger than their White counterparts, and younger adults are more likely to achieve hypertension control. We may want to compare outcomes among Black and White participants with a similar age (or age distribution) so that differences in age do not mask the effect of barriers that Black participants are more likely to face in managing their hypertension. Such barriers may include their greater likelihood of residence in neighborhoods with fewer healthy food stores, options for physical activity, and pharmacies to support medication adherence (Mueller et al., 2015). For this very reason, we would not want to similarly situate Black and White participants on socioeconomic status (SES), since the Black participants’ lower SES is a primary driver of these barriers and their worse hypertension control, especially if we believe that the opportunity to achieve hypertension control should not depend on one’s SES. Adjusting for SES would, in a sense, over-adjust the measure of racial disparity used to define the intervention effect. In essence, we may not wish to adjust for all covariates as ${\varvec{X}}$, but rather a subset ${\varvec{A}}$ that we designate as allowable, where another non-allowable subset ${\varvec{N}}$ is used to account for confounding or improve precision but not to adjust the disparity measure. The choice of what is allowable (if anything at all) is a complex but necessary choice and can be informed by ethical and justice-based frameworks.^2^ From here on, in our examples, we will assume that we have chosen age and gender as allowable ${\varvec{A}}$ and SES as non-allowable covariates ${\varvec{N}}$ for the reasons discussed above.

When we express the intervention effect $\tau$ on disparity as a contrast of the intervention effects across social groups, conditional on the same covariates as in (3), it resembles an interaction term that quantifies effect heterogeneity. On the risk difference (i.e., additive) scale,

4 $${\tau }^{RD}(x)$$

$$=\left(\mu \left(Z=1,R=1,x\right)-\mu \left(Z=0,R=1,x\right)\right)-\left(\mu \left(Z=1,R=0,x\right)-\mu \left(Z=0,R=0,x\right)\right)$$

The perspective of (4) shows us that the choice of what covariates are considered allowable (i.e., what is included in ${\varvec{X}}$ and thus used to define disparity) may impact the magnitude of the intervention effect on disparity. The intervention will reduce the racial disparity in hypertension control when its effect is, on average, greater among Black participants (Mackenbach & Gunning-Schepers, 1997). Ideally, the intervention achieves this by addressing barriers that are overrepresented among Black participants (Cooper et al., 2002). For example, persons who are adherent to antihypertensive medications are more likely to achieve hypertension control. If baseline adherence is lower among Black participants, we expect an intervention that improves adherence to be more effective in increasing hypertension control for this group (as more people in this group stand to benefit from the intervention). But if we similarly situate Black and White participants on baseline adherence, we expect this intervention to be equally effective across racial groups because the effects are compared between groups with similar baseline adherence. Supplemental Fig. 1 provides a more formal intuition based on analysis of a causal graph.

When applied researchers analyze intervention effects, especially those of multilevel interventions, they may need to adjust for many covariates that are implicated in disparity if they are imbalanced across intervention arms or if they are associated with study attrition. Imbalanced covariates may be likely to occur in practice with multilevel interventions when randomization occurs at the cluster level and there are few clusters. If we fail to adjust for certain covariates, we may have bias due to confounding or study attrition. But if we adjust through regression, we may overadjust the disparity measure and obscure the treatment effect on disparity as we discussed above. We need a modeling approach that can use a chosen set of allowable covariates ${\varvec{A}}$ (e.g., age and gender) to define the effect on the disparity in the outcome while using an auxiliary set of non-allowable covariates ${\varvec{N}}$ (e.g., SES) to address potential bias without overadjustment.

### Decision-Based Process Outcomes

At times we may wish to conduct exploratory analyses on process outcomes that involve medical decision-making. For example, we may wish to know how the intervention affects decisions $D$ to intensify antihypertensive medications as measured at follow-up (after hypertension control $Y$). But this raises questions about allowability. In Supplemental Table 2, we provide a hypothetical example, which we explain here. The Black-White difference in uncontrolled hypertension at follow-up is absent in the treatment arm (0%), and present in the control arm (40%). If we assess the intervention’s impact on the disparity in antihypertensive treatment intensification $D$ while ignoring hypertension control $Y$, we find no difference in the treatment arm $Z=1$ (0%), and higher antihypertensive treatment intensification among Black participants than White participants in the control arm $Z=0$ (8%). But, among those with the same level of hypertension control $Y$, there is no Black-White difference in $D$ in the treatment or control arm. What ostensibly was the intervention’s impact on difference in antihypertensive treatment intensification $D$ is entirely attributable to its impact on eliminating the Black-White difference in hypertension control $Y$.

This is a form of Simpson’s paradox (Simpson, 1951), where the correct choice (to account for $Y$) is driven by substantive rather than statistical considerations. From the standpoint of equity (Institute of Medicine Committee on Understanding & Eliminating Racial Ethnic Disparities in Health Care, 2003; Jackson, 2021), it makes sense to consider hypertension control $Y$, which reflects clinical need, as allowable for defining disparity (and thus effects on disparity) in antihypertensive treatment intensification $D$, a medical decision. However, we cannot simply adjust for $Y$ when measuring intervention effects on disparity in $D$ because $Y$ is a post-intervention variable and doing so may induce bias under more general settings than depicted in the hypothetical scenario of Supplemental Table 2. See, for example, the explanation based on a causal diagram in Supplemental Fig. 2. There, the allowable covariate itself may share a common cause with the outcome, and conditioning on the allowable without accounting for that common cause can lead to what is called collider-stratification bias (Cole & Hernán, 2002). We call this situation the “allowability dilemma” because the intervention effect’s interpretation may be difficult if one does not account for the post-intervention allowable criteria, and the effect estimate may be biased if one adjusts for criteria inappropriately.^3^ With intervention effects on disparity in decisions like $D$, we need to overcome the allowability dilemma by properly accounting for post-intervention allowables like $Y$.

Text Box 2 Our analytic approach draws from the causal inference literature. It addresses the first challenge by allowing analysts to estimate mean outcomes or proportions for each intervention arm (that account for potential confounding by measured covariates and potential selection-bias due to loss to follow-up), which can be combined to provide intervention effects on the additive or relative scales. It addresses the second challenge by balancing all covariates across intervention arms within each social group (e.g., to control for confounding) but only chosen covariates across social groups (to meaningfully define disparity). It addresses the third challenge by defining and estimating a novel direct effect that removes the impact of the intervention on disparity in the relevant decision criteria. The direct effect estimators account for confounding of the criteria and avoid introducing bias.

## Analytic Approach

We outline an analytic approach, based on the potential outcome framework, that overcomes the aforementioned challenges. Readers who are unfamiliar with this framework can find a brief review in the Supplemental Material.

We begin by considering estimation for the total intervention effect by weighting and by a sequential regression procedure known as g-computation (Snowden et al., 2011) and also as iterated conditional expectations (Wen et al., 2021). Weighting involves modeling the intervention assignment mechanism correctly and g-computation involves modeling the outcome process correctly. g-computation is more efficient (Ren et al., 2023) but weighting is designed-based and the weights, which can be constructed without knowledge of the outcomes, can be checked by evaluating covariate balance after weighting (Austin & Stuart, 2015; Jackson, 2016). Therefore, g-computation may be favored under limited sample size for estimation or power for hypothesis testing, whereas weighting may be favored to emphasize objectivity. For direct effects, the g-computation procedure does not require modeling the post-intervention allowable criteria, whereas the weighting procedure does. Both estimation approaches can provide effect estimates on the additive and relative scales. Unlike regression that adjusts for covariates (e.g., age), the approaches we propose allow for the intervention’s effect on disparity to be heterogeneous (e.g., to be more/less effective for various age groups) without having to formulate this form of heterogeneity in the modeling procedure.

Informally, the weighting and g-computation estimation approaches we will propose balance the allowable covariates ${\varvec{A}}$ (e.g., age and gender) and non-allowable covariates ${\varvec{N}}$ (e.g., SES) across intervention arms within each social group (to control for potential confounding by ${\varvec{A}}$ and ${\varvec{N}}$), while only balancing the allowables ${\varvec{A}}$ across social groups $R$ (i.e., across Black and White participants, to define meaningful effects on disparity). This form of separate balancing for allowables and non-allowables to meaningfully represent intervention effects on disparity represents a novel feature of our approach, which traditional causal estimands and their associated applications of weighting and g-computation estimators do not share. The total effects, which we consider first, are essentially intention-to-treat effects and are of most interest to practitioners who are interested in the total effect of the CC/SC versus SCP interventions as they were actually implemented in the treatment and control arms. Following this, we consider novel direct effects and their estimation by weighting and g-computation procedures. The direct effects are most useful for exploratory analyses for decision-based outcomes while avoiding the challenges described in the previous section.

### Definition of Total Effects

The potential outcome ${Y}_{i}^z$ is the outcome we would observe for individual under assigning that person to the intervention arm $Z=z.$ We omit the subscript $i$ to simplify notation. We denote the standardized^4^ average potential outcome among those in the social group $R=r$ under the intervention to set $Z$ to value $z$ as^5^:

5 $${\widetilde{\mu }}^{z}(r)={\sum }_{{\varvec{a}}}E\left[{Y}^{z}|R=r,{\varvec{A}}={\varvec{a}}\right]P({\varvec{A}}={\varvec{a}}|T=1)$$

On the risk difference (i.e., additive) scale, we define the intervention effect ${\widetilde{\tau }}^{RD}$, as

6 $${\widetilde{\tau }}^{RD}=\widetilde{\theta }(1)-\widetilde{\theta }(0)$$

where $$\widetilde{\theta }(z)={\widetilde{\mu }}^{Z=z}(R=1)-{\widetilde{\mu }}^{Z=z}(R=0)$$

The total effects can also be similarly defined for the relative scale which resembles (2).

The standardization of the allowables ${\varvec{A}}$ to a common within-sample standard distribution, denoted by $T=1$, balances them across social groups $R$ so that the definition of effect on disparity is meaningful. Whenever the intervention effect $\widetilde{\tau }$ is modified by the allowables ${\varvec{A}}$ on the chosen scale, the choice of standard population, denoted by $T=1$, will impact the magnitude of the effect. This choice can reflect inferential interests and value judgments. Reflecting inferential interests, the standard population represents membership in the entire trial sample when ${\widetilde{\tau }}^{RD}$ and ${\widetilde{\tau }}^{RR}$ represent sample average treatment effects on disparity (SATE-D), which is of interest if the intervention is to be applied to the entire trial sample. See the Supplemental Material for sample average effects of disparity among the treated (SATT-D). Reflecting value judgments, one may wish to center Black persons among the entire sample or among the treated by choosing them as the standard population in either case (Thurber et al., 2022). While this choice may seem to be an added analytic complexity, commonly used regression estimators that adjust for covariates can be represented as a form of standardization where the choice of standard population is usually data driven, opaque, and not connected to any actual population (Aronow & Samii, 2016). Being concrete about the standard population allows the analyst to choose their own inferential goals and makes their value judgments explicit.

### Estimation of Total Effects

Here we present two approaches (weighting and g-computation) for estimating the SATE-D given its relevance for our motivating example. We describe modifications for the SATT-D and for loss to follow-up in the supplementary material. The approaches rely on standard “identifying” assumptions (Hernán & Robins, 2020) to ensure that the average potential outcome can be estimated using the observed study data. A key assumption is that the effect of the intervention is unconfounded given the social group $R$, the allowables ${\varvec{A}}$ (e.g., age and gender), and non-allowables ${\varvec{N}}$ (e.g., SES), along with assumptions known as positivity and consistency, and overlap in the distribution of the allowables ${\varvec{A}}$ between each social group $R=r$ and the standard population $T=1$. These are described further in the Supplemental Material. If these assumptions hold, we can estimate each average potential outcome ${\widetilde{\mu }}^{z}(r)$ under each intervention arm $Z=z$ and racial group $R=r$.

To estimate ${\widetilde{\mu }}^{z}(r)$ for the SATE-D, the average effect oftreatment on disparity, by weighting we take those belonging to a particular social group $R=r$ in the treatment (or control) arm $Z=z$ and take a weighted average of their observed outcomes, using the weight:

7 $$\begin{aligned}{W}_{r,z}^{SATE-D}=&\frac{P(Z=z|R=r)}{P(Z=z|R=r,{\varvec{N}}={\varvec{n}},{\varvec{A}}={\varvec{a}})}\\&\times \frac{P(T=1|{\varvec{A}}={\varvec{a}})}{P(R=r|{\varvec{A}}={\varvec{a}})}\times \frac{P(R= r)}{P(T=1)}\end{aligned}$$

For the SATE-D, we choose the standard population $T=1$ among the entire trial sample. The first term of the weight controls for confounding of the intervention $Z$ by the allowables ${\varvec{A}}$ (e.g., age and gender) and non-allowables ${\varvec{N}}$ (e.g., SES) in each social group $R$ (e.g., Black and White participants), by making the treated and control arms of each social group $R$ comparable. The second and third terms serve to meaningfully define disparity. They do so for each arm $Z$ by balancing ${\varvec{A}}$ across $R$ in such a way that ${\varvec{A}}$ follows the standard distribution $P\left({\varvec{A}}={\varvec{a}}|T=1\right)$, the marginal distribution of ${\varvec{A}}$ in the entire study population who are members of the standard population denoted as $T=1$. Because the same standard distribution is used to balance ${\varvec{A}}$ across $R$ for both intervention arms, their comparability is preserved.

Because the weights are unknown, they must be estimated by modeling the assignment mechanism for the intervention $Z$ given the non-allowables ${\varvec{N}}$ and allowables ${\varvec{A}}$ within each social group $R$, modeling the patterning of $R$ given ${\varvec{A}}$, and patterning of $T$ given ${\varvec{A}}$. The predicted values from these models are then used to obtain the weights. For example, we could estimate the conditional probability $P(R=r |{\varvec{A}}={\varvec{a}})$ by fitting a logistic regression model for $R$ given ${\varvec{A}}$:

8 $$logit \bigl(P\left(R=1|\varvec{A}={\varvec{a}}\right)\!\bigl)= {\beta }_{0}+{\beta }_{A}^{\boldsymbol{^{\prime}}}{\varvec{A}}$$

For those in the marginalized group $R=1$ (e.g., Black participants), we use the coefficients to predict $\widehat{P}(R=1|{\varvec{A}}={\varvec{a}})$. For those in the privileged group $R=0$ (e.g., White participants), we obtain $\widehat{P}(R=0|{\varvec{A}}={\varvec{a}})$ by predicting $\widehat{P}(R=1|{\varvec{A}}={\varvec{a}})$ and obtaining the complement by subtracting from one. Similar strategies can be used to obtain the remaining components of (7).

To estimate ${\widetilde{\mu }}^{z}(r)$ for the SATE-D by g-computation, we propose a sequential regression and prediction procedure that relies on the re-expression of ${\widetilde{\mu }}^{z}(r)$ as an iterated expectation:

9 $${\widetilde{\mu }}^{z}{(r)}^{SATE-D}=E[\![E(E[Y|Z=z,R=r,{\varvec{N}}={\varvec{n}},{\varvec{A}}={\varvec{a}}]|R=r,{\varvec{A}}={\varvec{a}})|T=1]\!]$$

Here we present one algorithm for estimating (9). In step 1, we regress the outcome $Y$ on the allowables ${\varvec{A}}$ (e.g., age and gender) and the non-allowables ${\varvec{N}}$ (e.g., SES) among an intervention arm $Z=z$ and social group $R=r$, e.g., the generalized linear model:

10 $$g(E\left[Y|Z=z,R=r,{\varvec{N}}={\varvec{n}},{\varvec{A}}={\varvec{a}}\right])={\alpha }_{0}+{\alpha }_{1}^{\boldsymbol{^{\prime}}}{\varvec{A}}+{\alpha }_{2}^{\boldsymbol{^{\prime}}}{\varvec{N}}$$

where $g(\bullet )$ is some link function (e.g., identity [for linear regression], logistic). In step 2, among those in the entire social group $R=r$ (the same one chosen in step 1), we obtain the predicted values from the model fit in step 1 (e.g., from (10)), and we call those predictions $Q[1]$. In step 3, among the same social group $R=r$ used in steps 1 and 2, we regress the predictions $Q[1]$ on the allowables ${\varvec{A}}$ (e.g., age and gender), e.g., the generalized linear model:

11 $$g(E\left[Q\left[1\right]|R=r,{\varvec{A}}={\varvec{a}}\right])={\gamma }_{0}+{\gamma }_{1}^{\boldsymbol{^{\prime}}}{\varvec{A}}$$

In step 4, among the standard population denoted by $T=1$, which for the SATE-D is among the entire trial sample, we obtain predicted values from the model fit in step 3 (e.g., from (11)), and we call those predictions $Q[2]$. In step 5, among the standard population we take an average of these predicted values $Q[2]$, which estimates the standardized average potential outcome ${\widetilde{\mu }}^{z}(r)$. For standard errors and confidence intervals that account for the hierarchical structure of the data, we suggest a non-parametric, balanced, stratified cluster bootstrap procedure (Davison & Hinkley, 1997; Field & Welsh, 2007; Gleason, 1988; Huang, 2018; Ren et al., 2010). Bootstrap samples are formed by resampling clusters with replacement (at exactly the same rate per cluster) separately for treatment and control arms and retaining all observations within sampled clusters.

### Definition of Direct Effects

We discussed that the assessment of intervention effects on disparity in decision-based outcomes $D$ (e.g., treatment intensification) needs to properly account for certain post-intervention allowable criteria measured just before the decision (e.g., clinical need). This is necessary so that assessment of intervention effects on disparity in, say, treatment decision-making is not obscured by intervention effects on disparity in the criteria that inform the decision. To accomplish this, we define direct effects via two actions: (1) assign the treatment or control condition; (2) assign the post-intervention decision-relevant criteria in such a way that, within each arm, there is no disparity in the criteria. Although the second action is hypothetical, we could actually intervene to affect the decision-maker’s perception of the criteria (Tolbert & Jackson, 2024). Such strategies are often used in randomized audit studies (Bertrand & Duflo, 2017) designed to detect discrimination, e.g., in hiring decisions by assigning equal qualifications to resumes before passing them along to the hiring manager(s). Thus, the direct effect identifies the impact of the intervention on disparate treatment.

To define sample interventional direct effects of treatment on disparity (SITE-D), we introduce a different standardized average potential outcome^6^${\ddot{\mu }}^{z}\left(r\right)$ for a decision-based outcome $D$ at follow-up among those in the social group $R=r$ under a joint action to assign the intervention condition $Z$ to value $z$ and assign the values of the criteria ${\varvec{B}}$ (that are perceived by the decision-maker just before the decision $D$). Formally,

12 $${\ddot{\mu }}^{z}\left(r\right)= {\sum }_{{\varvec{a}}}E\left[{D}^{\left(z,{\varvec{G}}\right)}|R=r,{\varvec{A}}={\varvec{a}}\right]P({\varvec{A}}={\varvec{a}}|T=1)$$

where ${\varvec{G}}\equiv {\varvec{B}}\sim P({{\varvec{B}}}^{z}={\varvec{b}}|T=1)$ is an action to set ${\varvec{B}}$ to a value that was randomly drawn from a pre-specified distribution (Didelez et al., 2006; Geneletti, 2007; Muñoz & van der Laan, 2012) and $T=1$ denotes membership in a standard population within the entire trial sample.

Within each arm $Z=z$, the criteria ${\varvec{B}}$ (e.g., hypertension control at follow-up $Y$) are set by drawing their values from a counterfactual distribution obtained from the standard population $T=1$ after intervening to set $Z=z$ (e.g., to treatment or control). The distribution used to draw the assigned values for the criteria (the assigned distribution) is a counterfactual distribution under the treatment condition (when the intervention is set to treatment), or under the control condition (when the intervention is set to control). This has two implications. First, the direct effect captures effects of the intervention on decision-making through the criteria. But it does not capture effects of the intervention on disparity in the criteria. In this way, the effects of the intervention on disparity in decision-making (e.g., treatment intensification) are not obscured by effects of the intervention on disparity in the criteria (e.g., hypertension control).

On the risk difference (i.e., additive) scale, we define the direct effect ${\ddot{\tau }}^{RD}$, as

13 $${\ddot{\tau }}^{RD}=\ddot{\theta }(1)-\ddot{\theta }(0)$$

where $$\ddot{\theta }(z)={\ddot{\mu }}^{Z=z}\left(R=1\right)-{\ddot{\mu }}^{Z=z}\left(R=0\right)$$

Relative effects can also be defined to resemble (2). See the Supplemental Material for sample interventional direct effects of treatment on disparity among the treated (SITT-D).

The direct effect is “direct” in the sense that it captures a sample-level effect on disparity in healthcare decisions (e.g., made by providers) that are not due to the intervention’s effect on disparity in the distribution of allowable criteria used to inform those decisions. As a sample-level effect, it differs from the sort of sample average direct effects that are defined by averaging over individual-level direct effects (Didelez et al., 2006; Pearl, 2001; Robins & Greenland, 1992; Robins et al., 2022; Vanderweele et al., 2014).

### Estimation of Direct Effects

We propose weighting and g-computation procedures to estimate the SITE-D. Modifications for estimating the SITT-D and under loss to follow-up appear in the Supplementary Material. The approaches rely on the “identifying” assumptions (Hernán & Robins, 2020) (beyond those of the SATE-D), namely unconfoundedness, positivity, consistency, and overlap for the effect of the post-intervention ${\varvec{B}}$ allowables on the outcome $D$, and for the effect of the intervention $Z$ on the post-intervention allowables ${\varvec{B}}$, which we describe in the Supplemental Material. If these assumptions hold, we can estimate each average potential outcome ${\ddot{\mu }}^{z}(r)$ under each intervention arm $Z=z$ and racial group $R=r$.

To estimate $${\ddot{\mu }}^{z}(r)$$ for the SITE-D, the interventional direct effect on disparity, by weighting we can take those from a social group $$R=r$$ in the intervention (or control) arm $$Z=z$$ and take a weighted average of their observed outcomes (e.g., treatment intensification $$D)$$, with the following weight, which incorporates the weight (7) used to estimate the SATE-D:

14 $${W}_{r,z,{\varvec{b}}}^{SITE-D}=\frac{E[P({\varvec{B}}={\varvec{b}}|Z=z,T=1,{\varvec{N}}={\varvec{n}},{\varvec{A}}={\varvec{a}})|T=1]}{P({\varvec{B}}={\varvec{b}}|Z=z,R=r,{\varvec{N}}={\varvec{n}},{\varvec{A}}={\varvec{a}})}\times {W}_{r,z}^{SATE-D}$$

The first term of the weight (14) takes each social group $R=r$ within each arm $Z=z$ and shifts the actual distribution of the post-intervention criteria ${\varvec{B}}$ (e.g., hypertension control) to the assigned distribution. This distribution depends on their intervention condition, but not their social group. The denominator is the actual distribution of the post-intervention allowables ${\varvec{B}}$ given the non-allowables ${\varvec{N}}$ and the baseline allowables ${\varvec{A}}$ among those in the intervention arm $Z=z$ and social group $R=r$. Estimating it requires modeling the actual distribution of ${\varvec{B}}$ given ${\varvec{N}}$ and ${\varvec{A}}$ among those with $Z=z$ and $R=r$. To aid the exposition, we assume that ${\varvec{B}}$ is discrete with a tractable number of levels (e.g., hypertension control defined in stages I-IV (Whelton et al., 2018)) so that a multinomial logistic regression model is appropriate. The predicted values of this model serve as the weight’s denominator. The numerator is similar, except that it is defined among those in the standard population $T=1$ (rather than among the social group $R=r$) and is marginalized over the distribution of the allowables ${\varvec{A}}$ and non-allowables ${\varvec{N}}$ among the standard population $T=1$. Estimating it requires fitting another multinomial logistic regression model for ${\varvec{B}}$ (this time fit among the standard population $T=1$ in the arm $Z=z$) given $({\varvec{A}}$,${\varvec{N}})$ and then obtaining predicted values from this second model among the standard population. The next step is to fit an intercept-only multinomial logistic regression model for ${\varvec{B}}$ among the standard population. The predicted values from this final model are the weight’s numerator.

To estimate ${\ddot{\mu }}^{z}(r)$ for the SITE-D by g-computation, we build upon the g-computation procedure for the SATE-D (9). The procedure for the SITE-D adds a few preliminary steps, the output of which is plugged in as the initial outcome for the SATE-D procedure. It relies on the re-expression of ${\ddot{\mu }}^{z}(r)$ as an iterated conditional expectation:

15 $${\ddot{\mu }}^{z}{\left(r\right)}^{SITE-D}$$

$$=E[\![E(E\{E[D|Z=z,R=r,{\varvec{B}}={\varvec{b}},{\varvec{N}}={\varvec{n}},{\varvec{A}}={\varvec{a}}]|Z=z,T=1,{\varvec{N}}={\varvec{n}},{\varvec{A}}={\varvec{a}}\}|R=r,A={\varvec{a}})|T=1]\!]$$

In preliminary step (i), we regress the outcome $D$ on the baseline allowables ${\varvec{A}}$ (e.g., age and gender) and the non-allowables ${\varvec{N}}$ (e.g., SES) and the post-intervention allowables ${\varvec{B}}$ (e.g., hypertension control) among an intervention arm $Z=z$ and social group $R=r$, e.g.,

16 $$g\left(E\left[D|Z=z,R=r,{\varvec{B}}={\varvec{b}},{\varvec{N}}={\varvec{n}},{\varvec{A}}={\varvec{a}}\right]\right)={\eta }_{0}+{\eta }_{1}^{\boldsymbol{{\prime}}}{\varvec{A}}+{\eta }_{2}^{\boldsymbol{{\prime}}}{\varvec{N}}+{\eta }_{3}^{\prime}{\varvec{B}}$$

where $g(\bullet )$ is some link function (e.g., identity, logistic). In preliminary step (ii), among those in the standard population $T=1$ with $Z=z$, we obtain predicted values from the model fit in step (i) (e.g., from (16)), and call those predictions $Q[{\text{i}}]$. In preliminary step (iii), among those in the standard population $T=1$ with $Z=z$, we fit a weighted regression where the predicted values $Q[{\text{i}}]$ from step (ii) are the regressand and the allowables ${\varvec{A}}$ and non-allowables ${\varvec{N}}$ are the regressors, e.g.,

17 $$g(E\left[Q\left[{\text{i}}\right]|Z=z,T=1,{\varvec{N}}={\varvec{n}},{\varvec{A}}={\varvec{a}}\right])={\lambda }_{0}+{\lambda }_{1}^{\boldsymbol{^{\prime}}}{\varvec{A}}+{\lambda }_{2}^{\boldsymbol{^{\prime}}}{\varvec{N}}$$

fit the following weights,

18 $${\omega }_{z}^{SITE-D}=\frac{P(Z=z|T=1)}{P(Z=z|T=1,{\varvec{N}}={\varvec{n}},{\varvec{A}}={\varvec{a}})}$$

The weights (18) ensure that the average taken over the post-intervention allowables ${\varvec{B}}$**,** which occurs in the regression (17), is over the appropriate counterfactual distribution of ${\varvec{B}}$. In preliminary step (iv) we obtain predicted values from the model fit in preliminary step (iii), and call those predictions $Q[{\text{ii}}]$. This completes the preliminary steps. The final predictions $Q[{\text{ii}}]$ then serve as the starting outcome for the g-computation procedure described for the SATE-D (9).

For the estimation of direct effects through weighting or g-computation, we suggest the same non-parametric, balanced, stratified cluster bootstrap described for the estimation of total effects. The statistical performance of the weighting and g-computation approaches for the SATE-D and SITE-D is compared in a brief simulation study in the Supplemental material.

## Application

We applied our methods to the RICH LIFE project to examine the total effect of the intervention, which occurred for 1 year, on the Black-White disparity in hypertension control at 2 years’ follow-up. While the direct effect on treatment intensification is of interest, this outcome is not yet available. To assess the potential for confounding and selection-bias, we compared the distribution of baseline predictors of hypertension control and study attrition across intervention arms separately for each group (see Supplemental Table 3). We estimated the average potential outcomes and total effect on disparity (SATE-D) of the RICH LIFE intervention (treatment [CC/SC arm] versus control [SCP arm]) on hypertension outcomes coded as controlled (gain) and again as uncontrolled (shortfall) at 2 years’ follow-up. For each coding, we estimated effects as a prevalence difference and as a prevalence ratio. For the reasons discussed earlier (see “allowability”), we chose age and gender as allowable and Black participants as the standard population. To adjust for potential confounding and selection-bias, we chose baseline measures of marital status, educational attainment, employment, smoking, systolic blood pressure, and medication adherence as variables for adjustment as potential non-allowable confounders, using the weighting and g-computation estimators for total effects, which were adapted for right censoring (see Supplemental Material). To simplify the application, we excluded the four participants with missing covariate data and the 67 individuals who died after baseline but before hypertension control could be measured. We used the balanced non-parametric cluster bootstrap, stratified by intervention arm and the presence of each racial group (or not) within the practice site, to obtain 95% confidence intervals.

Table 1 reports the average potential outcomes and effect estimates by outcome coding type and analytic approach. The results suggest that the 1-year CC/SC intervention may reduce uncontrolled hypertension at 2 years among both racial groups. However, it appears that this potentially sustained effect was similar across racial groups, with no impact on the disparity on the additive or relative scale. This may reflect the higher prevalence of personal and structural barriers to achieving hypertension control among Black participants (Supplemental Table 3), which may have been exacerbated during the COVID-19 pandemic. The direction of effects was similar on both scales and by coding type.

## Discussion

Motivated by the design of the RICH LIFE Project, we proposed analytic strategies for evaluating effects of multilevel interventions on disparity in health and health-related decisions. These analytic strategies were provided for inference in the entire trial sample (SATE-D and SITE-D), as emphasized in the main text, or for inference on the treated sample (SATT-D and SITT-D), as provided in the Supplemental Material. Effects among the treated are relevant for non-randomized designs when the treatment condition may not be expanded to the control sites that did not receive it as part of the trial.

The proposed approach enables analysts to estimate average potential outcomes for each racial group under each intervention condition (treatment or control), thus providing results on both additive and relative scales. It also allows for analysts to separate the balancing of covariates across social groups (to measure disparity) from the balancing of covariates across intervention arms and loss to follow-up (to account for confounding and selection-bias). Because the analytic approach is flexible with respect to effect scale, and to how covariates are balanced (across social groups and/or intervention arms), it allows one to incorporate values regarding what is equitable in the distribution of health with respect to measuring intervention effects.

Standard approaches such as regression analysis do not offer this degree of flexibility in scale or in how covariates are balanced and may not align with analysts’ or stakeholders’ underlying value judgments regarding equity. Furthermore, the ability of our analytic approach to provide marginal counterfactual means is helpful in clearly describing the intervention effect, especially the potential impact of the intervention if applied to the entire trial sample or only the marginalized social group. Standard approaches also do not allow for the estimation of effects on decision-based process outcomes such as treatment decision-making.

The total effects (SATE-D and SATT-D) are related to other approaches (Howe et al., 2018; Jackson & VanderWeele, 2018; Lundberg, 2022; Naimi et al., 2016; VanderWeele & Robinson, 2014) that estimate effects of hypothetical interventions on outcomes contrasted across social groups. Among other ways, our approach differs by examining the total effect of an actual intervention on disparity with two intervention conditions (treatment and control) rather than a single condition, by examining the total effect on disparity among the those in treated arm (SATT-D), and by explicitly considering allowable covariates and how they are standardized.

Our contribution also includes novel population-level direct effects for decision-based outcomes (SITE-D and SITT-D) that have not been considered before. They differ from the controlled direct effect (Pearl; Robins & Greenland, 1992), natural direct effect (Pearl; Robins & Greenland, 1992), principal stratum direct effect (Frangakis & Rubin, 2002), randomized interventional analog direct effect (Didelez et al., 2006; Geneletti, 2007; Vanderweele et al., 2014), organic direct effect (Lok, 2016), generalized direct effect (Nguyen et al., 2020), interventional direct effect (Robins et al., 2022), and causal influence direct effect (Díaz, 2023) in that (i) they are defined at the population-level whereas the existing direct effects are defined at the individual-level and then averaged (ii) they only remove the impact of the intervention on disparity in an intermediate variable or its perception (the post-intervention allowable criteria) rather than removing all intervention effects through the intermediate variable. The novel direct effect estimands are designed for the purpose of measuring an intervention’s effect on disparity in decision-making rather than providing mechanistic insight. Therefore, we do not define a complimentary indirect effect as the difference between the total and direct effects. Thus, the novel direct effects (SITE-D and SITT-D) avoid the criticisms (Miles, 2022) of indirect effects based on stochastic actions (Didelez et al., 2006; Geneletti, 2007; Vanderweele et al., 2014) on the intermediate variable.

Our approach is not without limitations. First, we note that the effects on disparity among the treated (the SATT-D and SITT-D presented in the Supplemental Material) may also be identified by adaptations of a difference-in-difference approach (Caniglia & Murray, 2020), or more accurately, triple-difference estimators used in economics to study effects on disparity. In those settings, the baseline outcomes can in certain cases be leveraged to enable weaker assumptions about confounding (Caniglia & Murray, 2020; Tchetgen et al., 2023), but the details for our total and direct effects on disparity are saved for future work. Second, our focus was on intention-to-treat effects but investigators and stakeholders may be interested in per protocol effects (Rojas-Saunero et al., 2022) that account for varying degrees of adherence to the intervention condition at the patient-level or site level. Such effects are of great interest in implementation science and will be considered in future work. Third, our approach for decision-based outcomes was limited to a single decision at the end of follow-up. Future work will consider effects on the trajectory of decisions over follow-up to better summarize effects on disparate patterns of decision-making. Our results for the SITE-D and SITT-D assume that any confounding of post-intervention allowable criteria is through baseline covariates. This will also be relaxed in future work. Our sample size determination procedure is based on desirable precision (e.g., confidence interval width) which reflects a focus on estimation. While this focus has many advantages (Rothman & Greenland, 2018), many users and funding agencies may desire to see sample size based on power for hypothesis testing. We suspect that leveraging permutation-based tests (Good, 1994) may be useful in this regard and leave this for future work.

Our approach does carry design implications. Estimating the SATE-D and SATT-D requires investigators to consider what baseline covariates are relevant for measuring disparity, and what additional non-allowable covariates may be prognostic for the outcome so that they can be accounted for in the analysis when there is evidence of potential confounding or selection-bias. Estimating the SITE-D and SITT-D requires measuring any post-intervention criteria that may be relevant for decision-making. Ideally these criteria would be measured just before the decision-based outcome, along with any potential variables that might confound these criteria.

Our contribution represents a potentially powerful approach for evaluating effects of multilevel interventions on disparity. This approach is also applicable to evaluating the effects of single-level interventions. In the Supplementary Material we provide sample analytic R code and a sketch of a simulation-based procedure for sample size determination based on precision.

### Supplementary Information

Below is the link to the electronic supplementary material.

Supplementary file1 (PDF 471 KB)

## Data Availability

The data underlying this article will be shared on reasonable request to the corresponding author.

## Footnotes

1 The R, SAS, and Stata statistical software packages do have built-in routines to marginalize over parameter estimates and report risk differences and, with some effort, risk ratios, although these routines have the limitation of treating all covariates as “allowable” by default (SAS Institute Inc., 2018; Sjölander, 2018; Williams, 2012).

2 To our knowledge, a general, a systematic approach for choosing what is allowable versus non-allowable is not yet available. Discussion of helpful principles is given in Jackson (2021). We suspect that the choice will, at the least, depend on: (i) a theory of how marginalization gives rise to population-level outcomes that are worse among the marginalized group, and (ii) some sense of what variables are morally appropriate in their contribution to health outcomes or healthcare decisions. The choice will always be subjective and guided by moral arguments and theories of (in)justice. We view this as desirable because it centers arguments about inequity which encourages reflection and discussion. See also Cook et al. (2009). When the choice of what is allowable does not align with the investigator’s underlying judgments about equity, the discrepancy in results can be viewed as a form of bias, which can be quite large. See Chang et al. (2024) for a simulation study demonstrating the magnitude of the bias in a different context.

3 The allowability dilemma cannot be resolved by carrying out a regression analysis with non-allowable covariates as additional regressors (to eliminate bias from collider-stratification) because this may obscure effects on disparity.

4 The effect is defined by standardizing potential outcomes. For such an effect, the standardization of the potential outcome can be interpreted as the average potential outcome that would be observed in a study design where the eligible trial participants were first sampled (at baseline before the intervention to set $Z$ to $z$) in such a way so that the allowables ${\varvec{A}}$ of each group $R=r$ are distributed as in the within-sample standard population denoted by $T=1$ (which balances the allowables across groups). After such sampling, the intervention $Z$ is applied. For more conceptual and formal details underpinning this interpretation, see Jackson et al. (2022).

5 The parameter ${\widetilde{\mu }}^{z}(r)$ in equation (5) refers to potential outcomes where the parameter is indexed by the assigned intervention status $z$, denoted as a superscript, whereas $\mu (z,r)$ in equations (1) through (4) refers to observed outcomes indexed by the observed intervention status $z$, denoted parenthetically.

6 See the earlier footnote under total effects regarding the interpretation of a standardized potential outcome in the definition of the effect. In the case of the direct effect, it refers to the average potential outcome that would be seen in a design where eligible trial participants are first sampled at baseline before any application of any intervention and then, among those sampled and retained in the trial, the interventions $Z$ is applied, and the intervention $G$ is applied just before measurement of the outcome $D$. See Jackson et al. (2022) for further details on interpretation.
