# Supplementary material for: Evaluating Effects of Multilevel Interventions on Disparity in Health and Healthcare Decisions
Source: Prev Sci. 2024 Jun 22;25(Suppl 3):407–20. doi: 10.1007/s11121-024-01677-8 (PMC11239607; doi:10.1007/s11121-024-01677-8)
Supplement: Supplementary file 1 — Supplementary file1 (PDF 471 KB) [file 11121_2024_1677_MOESM1_ESM.pdf]

SUPPLEMENTAL MATERIAL FOR "EVALUATING EFFECTS OF MULTILEVEL INTERVENTIONS ON DISPARITY IN HEALTH AND HEALTHCARE DECISIONS"

John W. Jackson, Yea-Jen Hsu, Lauren C. Zalla, Kathryn A. Carson, Jill A. Marsteller, Lisa A. Cooper and the RICH LIFE Project Investigators

Table of Contents

Supplemental Figure	Page 2
Supplemental Tables	Page 3
Review of the Potential Outcomes Framework	Page 6
Informal Description of Assumptions	Page 7
Definition and Estimation of SATT-D and SITT-D	Page 8
Estimation under Study Attrition / Loss to Follow-up	Page 11
Summary of Cluster Bootstrap Procedure	Page 13
Simulation Procedure for Sample Size Determination	Page 15
Simulation Study of Proposed Estimators	Page 17
Proof of Results (*with formal statements of assumptions*)	Page 18
References	Page 27
Example Code	Page 28

Supplemental Figures and Legends

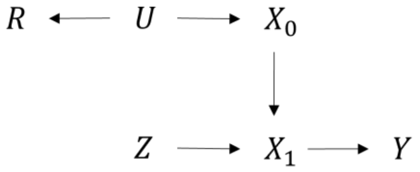

Supplemental Figure 1. Simplified causal diagram depicting effect measure modification of the intervention Z effect on hypertension control at follow-up by racial group membership R through a baseline covariate X_0 (e.g., baseline medication adherence) where it is assumed that Z causally interacts with X_0 (but not R) to produce X_1 (e.g., medication adherence during follow-up) and Y (e.g., subsequent hypertension control). In this oversimplified causal structure, R is an effect measure modifier by common cause (VanderWeele & Robins, 2007) because of its relationship with X_0 through unmeasured variable U (e.g., experiences of structural racism (Bailey et al., 2017)). When the analysis balances the distribution of X_0 across levels of R through some form of design or analytic adjustment (e.g., restriction, regression, standardization) then R no longer has an association with X_0 and no longer serves as an effect measure modifier in the analytic sample. (VanderWeele & Robins, 2007) In this scenario, the intervention Z may be more effective in changing Y among those with $R = 1$ when ignoring X_0, but after adjusting for X_0 it is equally effective in changing Y among those with $R = 1$ and $R = 0$ in the analytic sample. While more elaborate causal diagrams have nuances, the principle that adjustment choices can impact estimates of intervention effect on disparity remains.

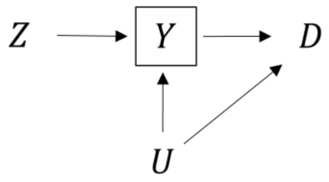

Supplemental Figure 2. Simplified causal diagram depicting collider-stratification bias for the effect of the intervention Z (e.g., treatment versus control) on the decision-based outcome D (e.g., treatment intensification) when the analysis conditions on the relevant criteria Y (e.g., hypertension control). Race R is omitted to simplify the diagram and the exposition. The box around Y indicates that the analysis conditions on Y which opens a non-causal pathway of association Z →⍰← $U \rightarrow D$ that produces a spurious association between the intervention arm Z and the decision-based outcome D.

Supplemental Tables

sTable 1. Hypothetical Data in a Pragmatic Trial to Demonstrate the Impact of Scale on Intervention Effects

	Proportion with Controlled Hypertension at follow-up (Y=1)	
	White (R=0)	Black (R=1)
Control arm (Z=0)	80	70
Treatment arm 1 (Z=1)	55	45
Treatment arm 2 (Z=2)	64	56

This is an adaptation of the hypothetical experiment proposed by Asada (Asada, 2010) and extended by Kjellsson et al. (Kjellsson et al., 2015) where the initial condition is represented by the control arm, the red pill represented by treatment arm 1, and the blue pill represented by treatment arm 2, and the original outcome of life expectancy is replaced by the proportion with controlled hypertension (an attainment). Relative to the control condition, the intervention in treatment arm 1 has no effect on the additive disparity, (45-55)-(70-80)=0, but increases the relative disparity (45/55)/(70/80)=.94. Whereas the intervention in treatment arm 2 increases the additive disparity (56-64)-(70-80)=2, but has no effect on the relative disparity (56/64)/(70/80)=1. When the outcome is coded as a shortfall (uncontrolled hypertension), the absolute values of the additive disparity do not change. However, now the intervention in treatment arm 1 decreases the relative disparity to a different extent than was increased before under attainment coding, (55/45)/(30/20)=.81, and the intervention in arm 2 now decreases the relative disparity to the same amount as in arm 1, (44/36)/(30/20)=.81.

sTable 2. Hypothetical Data in a Pragmatic Trial to Demonstrate the "Allowability Dilemma" for Decision-Based "Process" Outcomes

	Controlled Hypertension (Y=0)		Uncontrolled Hypertension (Y=1)	
	Dose Intensified (No) (D=0)	Dose Intensified (Yes) (D=1)	Dose Intensified (No) (D=0)	Dose Intensified (Yes) (D=1)
Control arm (Z=0)				
White (Z=0)	800	0	160	40
Black (R=1)	400	0	480	120
Treatment arm (Z=1)				
White (R=0)	400	0	480	120
Black (R=1)	400	0	480	120

To simplify this example, we assume that the effect of hypertension control at follow-up Y on subsequent dose change at follow-up D is unconfounded. This makes it appropriate estimate the difference in D while stratifying on Y, as doing so in this case induces no bias. In this example, the additive Black-White difference in Y (counting uncontrolled hypertension as the outcome) is absent among the treatment arm $Z = 1$, 600/1000 – 600/1000 = 0, and present among the control arm $Z = 0$, 600/1000 – 200/1000 = 400/1000. Meanwhile, the additive Black-White difference in D (counting a dose change as the outcome) while ignoring Y is absent among the treatment arm $Z = 1$, (0+120)/1000 – (0+120)/1000 = 0, and Black participants have a higher proportion of dose change than White participants among the control arm $Z = 0$, (0+120)/1000 – (0+40)/1000 = 80/1000. However, the additive Black-White difference in D while accounting for Y is absent for the treatment arm $Z = 1$, 0+(120/600 – 120/600) * 1200/2000 = 0, as well as for the control arm $Z = 0$, 0+(120/600 – 40/200) * 800/2000 = 0. In summary, while the intervention decreases the Black-White difference in hypertension control Y, its effect on antihypertensive dose changes at follow-up D depends on whether or not hypertension control at follow-up Y is accounted for. When it is ignored, the intervention appears to increase antihypertensive treatment intensification more often for Black participants. Whereas when hypertension control at follow-up Y is accounted for, the intervention has no effect on the difference in antihypertensive treatment intensification.

sTable 3. Selected Baseline Characteristics of RICH LIFE Project Participants by Race and Intervention arm				
	Black Participants (R=1)		White Participants (R=0)	
	Treatment (Z=1) N=579	Control (Z=0) N=465	Treatment (Z=1) N=272	Control (Z=0) N=332
Baseline SBP, mean (sd)	153.8 (13.2)	153.1 (12.8)	150.74 (10.8)	150.2 (10.3)
Baseline DBP, mean (sd)	88.4 (12.7)	87.2 (11.8)	82.82 (12.3)	80.3 (10.8)
Age, mean (SD)	57.6 (11.2)	58.4 (11.5)	65.1 (11.1)	65.1 (12.1)
Female, N (%)	372 (64.2)	295 (63.4)	148 (54.4)	162 (48.8)
Married / Cohabitating, N (%)	206 (35.6)	163 (35.3)	169 (62.1)	194 (58.4)
Educational Attainment, N (%)				
<High School	143 (24.8)	72 (15.5)	17 (6.3)	18 (5.4)
High School	285 (49.4)	254 (54.6)	124 (45.8)	137 (41.4)
Some College	55 (9.5)	60 (12.9)	47 (17.3)	45 (13.6)
≥Bachelors	94 (16.3)	79 (17.0)	83 (30.6)	131 (39.6)
Employment, N (%)				
Employed	215 (37.3)	180 (39.0)	108 (39.9)	131 (39.5)
Unemployed	219 (38.0)	149 (32.3)	45 (16.6)	47 (14.2)
Retired	143 (24.8)	132 (28.6)	118 (43.5)	154 (46.4)
Smoking status, N (%)				
Current	176 (30.4)	135 (29.0)	38 (14.0)	52 (15.7)
Former	127 (21.9)	112 (24.1)	93 (34.2)	120 (36.1)
Never	276 (47.7)	218 (46.9)	141 (51.8)	160 (48.2)
Medication adherence, N (%)	253 (43.7)	194 (41.7)	174 (64.0)	236 (71.1)
Community violence, mean (sd)	0.9 (1.3)	0.9 (1.3)	0.4 (0.9)	0.5 (1.0)
Community disorder, mean (sd)	3.5 (3.8)	3.2 (3.9)	1.5 (2.3)	1.7 (2.6)
Health literacy, mean (sd)	2.2 (0.5)	2.1 (0.5)	2.1 (0.5)	2.0 (0.5)
Study attrition*, mean (sd)	179 (30.9)	141 (30.3)	24 (8.8)	32 (9.6)

Abbreviations: SBP systolic blood pressure, DBP diastolic blood pressure, BPC blood pressure control
*Study attrition assessed cumulatively at two year follow-up

Review of the Potential Outcomes Framework

We briefly review the potential outcomes framework (Neyman, 1923; Robins, 1986; Rubin, 1974) which underpins our proposed analytic approach. The potential outcome Y_i^z for an individual *i* represents the level of hypertension control Y_i (i.e., the outcome) that would be seen after assigning that person to the arm $Z_i = z$. The causal effect for each person *i* is the contrast of potential outcomes under assigning that person to treatment $Z_i = 1$ (e.g., CC/SC) versus control $Z_i = 0$ (e.g., SCP), i.e., $Y_i^1 - Y_i^0$. Because we only observe potential outcomes under treatment Y_i^1 for those assigned to treatment $Z = 1$, and potential outcomes under control Y_i^0 for those assigned to control $Z = 0$, we cannot compute effects for any individual. But we can consider sample-level causal effects, such as the sample average treatment effects (SATE) on the additive scale $E[Y^1] - E[Y^0]$ or the relative scale $E[Y^1]/E[Y^0]$ (dropping the subscript *i* indexing individuals to simplify the notation). If we consider those who were assigned treatment $Z = 1$ and compare their observed outcomes to their potential outcomes if assigned to control, we have the sample average effect of treatment among the treated (SATT), e.g., $E[Y|Z = 1] - E[Y^0|Z = 1]$ on the additive scale, and $E[Y|Z = 1]/E[Y^0|Z = 1]$ on the relative scale.

Although we do not observe the average potential outcome $E[Y^z]$ for the entire sample under assignment to treatment or control, we can identify it under certain assumptions, informally: 1) exchangeability: had the intervention assignment been swapped (so that those assigned to treatment were instead assigned to control) we would obtain the same effect estimate; 2) positivity: we have persons represented among both arms; 3) consistency: a potential outcome for any individual under a given treatment is well defined. Similarly we can use these asusmptions to identify the average potential outcome among the treated $E[Y^z|Z = 1]$. These assumptions are all expected to hold in an ideal randomized controlled trial.

In observational settings, where intervention assignment is not randomized, the assumptions may hold after conditioning on the covariates $\boldsymbol{X}$. In our example of a cluster randomized trial with few clusters, even though assignment is randomized, we nonetheless may need to condition on covariates $\boldsymbol{X}$ when they are imbalanced across intervention arms to achieve conditional exchangeability. (M.A. Hernán & Robins, 2020) Covariate adjustment can also be used to improve precision. (Colantuoni & Rosenblum, 2015)

Informal Description of Assumptions (see proofs starting on page 18 for formal statements)

Total Effects (SATE-D and SATT-D)

1) Members of each social group R (e.g., Black and White participants) are observed at each level of the allowables within the standard population, i.e., "common support", so the allowables $\boldsymbol{A}$ (e.g., age and sex) can be balanced and meaningful effects on disparity can be defined.

2) The effect of the intervention Z on the outcome Y hypertension control are unconfounded given the allowables $\boldsymbol{A}$ (e.g., age and sex) and non-allowables $\boldsymbol{N}$ (e.g., SES), separately for each social group R, i.e., "no unmeasured confounding" or "conditional exchangeability."

3) Within each social group $R = r$, participants in each intervention arm Z are represented within each level of the allowable $\boldsymbol{A}$ and non-allowable $\boldsymbol{N}$ covariates, i.e., "positivity."

4) The same outcomes would be observed if persons happened to select into their intervention arm rather than it being assigned to them (i.e., "consistency"). In cluster randomized trials, consistency may be violated under "interference" if one person's assignment affects the potential outcomes another person under the same or alternate assignment. However, the potential outcomes for the SATE-D and SATT-D remain well defined under "partial interference" (Sobel, 2006) wherein one person's assignment can only affect the potential outcomes of another person who, being in the same cluster, receives the same assignment. This partial interference assumption is met by the design of our motivating example, where intervention assignments were determined at the cluster level.

Note that in our example, which is a cluster randomized trial, assumption 2 may be required because the intervention is assigned at the cluster level and there are few clusters, possibly resulting in "chance confounding" (Greenland & Mansournia, 2015) by (non)-allowables.

Direct Effects (SITE-D and SITT-D)

1) – 4) above replacing Y (e.g., hypertension control) with D (e.g., treatment intensification).

5) Within each intervention arm $Z = z$, members of each social group R (e.g., Black and White participants) are observed at each level of the post-intervention allowables $\boldsymbol{B}$ within the standard population, i.e., "common support".

6) The effect of the post-intervention allowable criteria $\boldsymbol{B}$ (e.g. hypertension control Y) on the decision-based outcome D (e.g., treatment intensification) is unconfounded given the baseline allowables $\boldsymbol{A}$ (e.g., age and sex) and the non-allowables $\boldsymbol{N}$ (e.g., SES) among each intervention arm $Z = z$ and social group $R = r$, i.e., "exchangeability" (Miguel A. Hernán & Robins, 2006).

7) Within each social group $R = r$ and intervention arm $Z = z$, participants in each level of the post-intervention allowables $\boldsymbol{B}$ are represented within each level of the allowable $\boldsymbol{A}$ and non-allowable covariates, i.e., "positivity" (Zivich et al., 2022).

8) The same outcomes would be observed if persons happened to select into their observed level of $\boldsymbol{B}$ rather than it being assigned, i.e., "consistency" (VanderWeele, 2009).

9) We also need conditional exchangeability, positivity, and consistency assumptions for the effect of the intervention on Z on the post-intervention allowables $\boldsymbol{B}$ to identify the hypothetical interventional distribution for $\boldsymbol{B}$ used to define the direct effect (analogous to assumptions 2), 3), and 4) replacing Y with $\boldsymbol{B}$).

Definition of the SATT-D

We can define the potential outcome mean $\tilde{\mu}^z(r)$ among the treated arm:

$$\tilde{\mu}^z(r) = \sum_{\boldsymbol{a}} E[Y^z|Z=1, R=r, \boldsymbol{A}=\boldsymbol{a}]\, P(\boldsymbol{A}=\boldsymbol{a}|Z=1, T=1) \quad (1)$$

where $T = 1$ denotes membership in a within-sample standard population among the treated

The standard population represents all persons in the treated arm $Z = 1$ when $\tilde{\tau}^{RD}$ and $\tilde{\tau}^{RR}$ represent sample average treatment effects on disparity among the treated (SATT-D), which may be of interest when one only wants to know the effect of the intervention among those in the treated CC/SC arm if they had received the SCP condition. In our example, the SATT-D would be relevant if RICH LIFE had used a non-randomized design[1] and the CC/SC intervention was to be implemented among the clinics that received it in the trial.

On the risk difference (i.e., additive) scale, we define the treatment effect $\tilde{\tau}^{RD}$, as:

$$\tilde{\tau}^{RD} = \tilde{\theta}(1) - \tilde{\theta}(0) \quad (2)$$

where $\tilde{\theta}(z) = \tilde{\mu}^{Z=z}(R=1) - \tilde{\mu}^{Z=z}(R=0)$

Estimation of the SATT-D

Weighting

To estimate $\tilde{\mu}^z(r)$ for the SATT-D by weighting, the average effect of treatment on disparity among the treated, we modify the approach for the SATE-D by: 1) choosing the standard population among the treated population $Z = 1$; 2) using the modified weights:

$$W_{r,z}^{SATT-D} = \frac{P(Z=1|R=r,\boldsymbol{N}=\boldsymbol{n},\boldsymbol{A}=\boldsymbol{a})}{P(Z=z|R=r,\boldsymbol{N}=\boldsymbol{n},\boldsymbol{A}=\boldsymbol{a})} \times \frac{P(T=1,Z=1|\boldsymbol{A}=\boldsymbol{a})}{P(R=r,Z=1|\boldsymbol{A}=\boldsymbol{a})} \times \frac{P(R=r,Z=z)}{P(T=1,Z=1)} \quad (3)$$

Then, the model fit (e.g. logistic regression) and used to predict $R = 1$ (to obtain $P(R = 1)$ as would be done in estimating the SATE-D) would be replaced by a multinomial logistic regression model to predict the category that represents membership in a group jointly defined by social group and intervention arm, i.e., $(R = r, Z = z)$ to obtain $P(R = r, Z = z)$. A similar approach would be used to obtain the denominator of the last term. Note that for those in the treated arm $Z = 1$, the weight $W_{r,z}^{SATT-D}$ (3) reduces to the second and third terms.

G-computation

To estimate $\tilde{\mu}^z(r)$ for the SATT-D by g-computation, we can take a very similar approach as described in the main text for the SATE-D. The approach relies on the fact that $\tilde{\mu}^z(r)$ under the SATT-D can also be expressed as an iterated expectation:

$$\tilde{\mu}^z(r)^{SATT-D} = E[\![E(E[Y|Z=z,R=r,\boldsymbol{A}=\boldsymbol{a},\boldsymbol{N}=\boldsymbol{n}])|Z=1,R=r,\boldsymbol{A}=\boldsymbol{a})|Z=1,T=1]\!] \quad (4)$$

[1] In non-randomized designs the treated and control arms are more likely to differ in the distribution of covariates (some of which may serve to modify the effect of the intervention) so that the effect that would be seen among those in the entire trial sample differs from the effect that would be seen among those in the treated arm.

Essentially, the same five step algorithm used for the SATE-D can be used with a few changes. In the step 2 the predictions $Q[1]$ from the model fit in step 1 are made among the social group $R = r$ in the treatment arm $Z = 1$. We also choose the standard population $T = 1$ from those in the treatment arm $Z = 1$, so that in step 4 the predictions $Q[2]$ from the model fit in step 3 are made among the standard population $T = 1$ in the treatment arm $Z = 1$, and the final average of $Q[2]$ in step 5 is taken among the standard population $T = 1$ in the treatment arm $Z = 1$. Note that when estimating $\tilde{\mu}^z(r)$ under treatment $z = 1$, we skip steps 1 and 2 and use the observed outcome Y (e.g., hypertension control) as the dependent variable in the model fit in step 3.

Definition of the SITT-D

As with total effects, we must decide whether we are interested in direct effects among the entire trial sample or only among the treated. In the main text, the entire trial population was used to define the standard population when $\ddot{\tau}^{RD}$ and $\ddot{\tau}^{RR}$ represent sample interventional direct effects of treatment on disparity (SITE-D) for the entire trial sample. Alternatively, we may define the average potential outcome means $\ddot{\mu}^z(r)$ as among the treatment arm $Z = 1$:

$$\ddot{\mu}^z(r) = \sum_{\boldsymbol{a}} E\left[D^{(z,\boldsymbol{G})} \middle| Z = 1, R = r, \boldsymbol{A} = \boldsymbol{a}\right] P(\boldsymbol{A} = \boldsymbol{a} | Z = 1, T = 1) \qquad (5)$$

where $\boldsymbol{G} \equiv \boldsymbol{B} \sim P(\boldsymbol{B}^z = \boldsymbol{b} | Z = 1, T = 1)$ is an action (Didelez et al., 2006; Geneletti, 2007; Muñoz & van der Laan, 2012) to set $\boldsymbol{B}$ to a value that was randomly drawn from a pre-specified distribution

and $T = 1$ denotes membership in a within-sample standard population among the treated

When this is done, the standard population is chosen among the treatment arm $Z = 1$ and $\ddot{\tau}^{RD}$ and $\ddot{\tau}^{RR}$ represent sample interventional direct effects of treatment on disparity among the treated (SITT-D). The SITE-D is relevant for understanding effects on decision-based outcomes among the entire trial sample, and the SITT-D for effects among the treated in non-randomized designs.

On the risk difference (i.e., additive) scale, we define the treatment effect $\ddot{\tau}^{RD}$, as:

$$\ddot{\tau}^{RD} = \ddot{\theta}(1) - \ddot{\theta}(0) \qquad (6)$$

where $\ddot{\theta}(z) = \ddot{\mu}^{Z=z}(R = 1) - \ddot{\mu}^{Z=z}(R = 0)$

Estimation of the SITT-D

Weighting

To estimate $\ddot{\mu}^z(r)$ for the SITT-D by weighting, we can use a similar weight as in the main text:

$$W_{r,z,\boldsymbol{b}}^{SITT-D} = \frac{E[\![P(\boldsymbol{B}=\boldsymbol{b}|Z=z,T=1,\boldsymbol{N}=\boldsymbol{n},\boldsymbol{A}=\boldsymbol{a})|Z=1,T=1]\!]}{P(\boldsymbol{B}=\boldsymbol{b}|Z=z,R=r,\boldsymbol{N}=\boldsymbol{n},\boldsymbol{A}=\boldsymbol{a})} \times W_{r,z}^{SATT-D} \qquad (7)$$

The only difference is that for the SITT-D the standard population is defined among the treatment arm $Z = 1$ (rather than among the entire trial sample as it was for the SITE-D), and the weight incorporates (3) used to estimate the SATT-D.

G-computation

To estimate $\ddot{\mu}^z(r)$ for the SITT-D by g-computation, we can take a very similar approach which relies on the fact that $\tilde{\mu}^z(r)$ under the SATT-D can also be expressed as an iterated expectation:

$$\ddot{\mu}^z(r)^{SITT-D} \\ = E⟦E(E\{E[D|Z=z,R=r,\boldsymbol{B}=\boldsymbol{b},\boldsymbol{N}=\boldsymbol{n},\boldsymbol{A}=\boldsymbol{a}]|Z=z,T=1,\boldsymbol{N}=\boldsymbol{n},\boldsymbol{A}=\boldsymbol{a}\}|Z=1,R=r,A=\boldsymbol{a})|Z=1,T=1⟧ \quad (8)$$

The procedure is the same as for the SITE-D with two minor changes. First, the standard population is chosen among the treated arm. Second, the weights used in preliminary step (iii) are replaced with:

$$\omega_z^{SITT-D} = \frac{P(Z=1|T=1,\boldsymbol{N}=\boldsymbol{n},\boldsymbol{A}=\boldsymbol{a})}{P(Z=z|T=1,\boldsymbol{N}=\boldsymbol{n},\boldsymbol{A}=\boldsymbol{a})} \times \frac{P(T=1,Z=z)}{P(T=1,Z=1)} \quad (9)$$

Then the final predictions Q[ii] then serve as the starting outcome for the g-computation procedure described for the SATT-D (4).

Estimation under Study Attrition / Loss to Follow-up

Assumptions and Estimation for SATE-D and SATT-D (see assumptions in Proofs on page 18)

For identification of the SATE-D and the SATT-D under study attrition C (1=lost to follow-up, 0=otherwise), we modify the conditional exchangeability, positivity, and consistency statements to be with respect to intervention status $Z = z$ and not being lost to follow-up $C = 0$ jointly. The assumptions we present here are with respect to our proposed design where covariates are only measured at baseline and at follow-up. Weaker assumptions are possible. For example, one may make sequential assumptions, first for Z and then for C. Such assumptions allow for the use of covariates measured in between baseline and follow-up. However, given the motivating example of our prosed design, and for brevity, we do not present them here.

When the assumptions hold, we can extend the weighting and g-computation estimators for total effects to remove the selection bias from differential loss to follow-up. The weighting approach is modified by multiplying the weights $W_{r,z}$ for the SATE-D and SITT-D by the component:

$$W_c = \frac{P(C=0|Z=z,R=r)}{P(C=0|Z=z,R=r,\boldsymbol{N}=\boldsymbol{n},\boldsymbol{A}=\boldsymbol{a})} \qquad (10)$$

To modify the g-computation algorithm, the innermost expectations are conditioned on not being lost to follow-up $C = 0$. Thus, the algorithm changes by simply fitting the model in step 1 among those not lost to follow-up rather than irrespective of loss to follow-up, while the remaining steps are carried out irrespective of loss to follow-up as described in the main text and above.

Assumptions and Estimation for SITE-D and SITT-D (see assumptions in Proofs on page 19)

In addition to the modified assumptions described above for the SATE-D and SATT-D, we require additional assumptions regarding the post-intervention allowables. First, the conditional exchangeability, positivity, and consistency assumptions required to identify the interventional distribution of the post-intervention allowables are to be made with respect to the intervention status $Z = z$ and not being lost to follow-up $C = 0$ jointly. Second, the conditional exchangeability, positivity, and consistency assumptions conditions needed to identify the effect of the post-intervention allowables on the treatment-decision-outcome D are conditional on not being lost to follow-up $C = 0$.

When these assumptions hold, we can extend the weighting and g-computation estimators for direct effects to remove the selection bias from differential loss to follow-up. The weighting approach is modified by incorporating the component W_c (10) above and by conditioning the probability statements in the first term of (7) above on being not lost to follow-up. Specifically, for the SITE-D:

$$W_{r,z,\boldsymbol{b},\boldsymbol{c}^*}^{SITE-D} = \frac{E[\![P(\boldsymbol{B}=\boldsymbol{b}|C=0,Z=z,T=1,\boldsymbol{N}=\boldsymbol{n},\boldsymbol{A}=\boldsymbol{a})|T=1]\!]}{P(\boldsymbol{B}=\boldsymbol{b}|C=0,Z=z,R=r,\boldsymbol{N}=\boldsymbol{n},\boldsymbol{A}=\boldsymbol{a})} \times W_c \times W_{r,z}^{SATE-D} \qquad (11)$$

For the SITT-D:

$$W_{r,z,\boldsymbol{b_k},\boldsymbol{c}^*}^{SITT-D} = \frac{E[\![P(\boldsymbol{B}=\boldsymbol{b}|C=0,Z=z,T=1,\boldsymbol{N}=\boldsymbol{n},\boldsymbol{A}=\boldsymbol{a})|Z=1,T=1]\!]}{P(\boldsymbol{B}=\boldsymbol{b}|C=0,Z=z,R=r,\boldsymbol{N}=\boldsymbol{n},\boldsymbol{A}=\boldsymbol{a})} \times W_c \times W_{r,z}^{SATT-D} \qquad (12)$$

Modifying the g-computation algorithm for the SITE-D or the SITT-D involves two changes. First, the innermost expectations are conditioned on not being lost to follow-up $C = 0$. Thus, the algorithm changes by simply fitting the model in preliminary step (i) among those not lost to

follow-up while the remaining preliminary steps are carried out irrespective of being lost to follow-up, as described in the main text and above. Second, in preliminary step (iii), the weights used to fit the weighted regression model are modified by multiplying the original weights ω_z by following term which accounts for loss to follow-up in preliminary steps (ii) and (iii):

$$\omega_C = \frac{P(C=0|Z=z,T=1)}{P(C=0|Z=z,T=1,\boldsymbol{N}=\boldsymbol{n},\boldsymbol{A}=\boldsymbol{a})} \tag{13}$$

Then, the final predictions Q[ii] from preliminary step (iii) are fed into the G-computation procedure for the SATE-D described in the main text or SATT-D in the Supplemental Material (i.e., the algorithm that assumes censoring is absent, because censoring has been addressed in the preliminary steps (i)-(iv)).

Summary of Clustered Bootstrap Procedure

Bootstrap Sampling Matrix

The non-parametric bootstrap (Efron & Tibshirani, 1986) is a re-sampling procedure that provides measures of uncertainty such as confidence intervals. The clustered bootstrap (Field & Welsh, 2007; Huang, 2018; Ren et al., 2010) is an extension of this approach to hierarchical data. In one version, bootstrap samples are formed by randomly sampling with replacement at the highest cluster level, retaining all observations within sampled clusters. The balanced bootstrap, introduced by (Davison et al., 1986), can improve the bootstrap's efficiency.

The balanced cluster bootstrap can be implemented by creating an initial $\mathcal{M} \times \mathcal{N}$ matrix (denoted as $\boldsymbol{\Omega}^{initial}$) with $\mathcal{M}$ rows whose indices uniquely track the cluster identification numbers (e.g., 1 ... $\mathcal{M}$), and $\mathcal{N}$ columns whose indices uniquely track the bootstrap samples (e.g., 1 ... $\mathcal{N}$). The following procedure transforms this starting matrix $\boldsymbol{\Omega}^{initial}$ into a new randomly sorted matrix $\boldsymbol{\Omega}^{sorted}$ (also of dimension $\mathcal{M} \times \mathcal{N}$) where for each row, the cluster identification numbers have been randomly sorted. The sorting ensures that across bootstrap samples, each cluster identification number appears exactly $\mathcal{N}$ times, making it balanced.

The following procedure, adapted from Deen and de Rooij (2020) and Gleason (1988) transforms the starting matrix $\boldsymbol{\Omega}^{initial}$ into the randomly sorted matrix $\boldsymbol{\Omega}^{sorted}$.

1. Transform $\boldsymbol{\Omega}^{initial}$ into a vector $\boldsymbol{\psi}^{initial}$ of dimension $1 \times \mathcal{O}$ where the length of the vector is equal to the product of $\mathcal{M}$ (the number of rows in $\boldsymbol{\Omega}^{initial}$) times $\mathcal{N}$ (the number of columns of in $\boldsymbol{\Omega}^{initial}$).
2. Generate a second vector $\boldsymbol{\psi}^{index}$ of the same dimension as $\boldsymbol{\psi}^{initial}$ (i.e., $1 \times \mathcal{O}$) where each element is a random draw from a uniform distribution $\mathbb{U}_{[min,max]}$. Any choice for min and max (the distribution's minimum and maximum values) may be used.
3. Sort the vector $\boldsymbol{\psi}^{index}$ according to increasing (or decreasing) value.
4. Use $\boldsymbol{\psi}^{index}$ to sort $\boldsymbol{\psi}^{initial}$, and rename the resulting vector $\boldsymbol{\psi}^{sorted}$.
5. Create an empty matrix $\boldsymbol{\Omega}^{sorted}$ of dimension $\mathcal{M} \times \mathcal{N}$. Then, by each column, fill the matrix with the values of $\boldsymbol{\psi}^{sorted}$.

Each column of the matrix $\boldsymbol{\Omega}^{sorted}$ provides the appropriate vector of cluster identification numbers to draw a bootstrap sample.

Stratifying the Bootstrap Sampling Matrix by Intervention Arm Z and Group Composition L

To ensure that clusters assigned to each intervention arm (treated $Z = 1$, control $Z = 0$) are represented within each bootstrap sample, so that an intervention effect can be calculated within each bootstrap sample, Steps 1 through 5 can be carried out separately for clusters in the treatment $Z = 1$ and control arm $Z = 0$. This produces two matrices $\boldsymbol{\Omega}^{sorted}_{Z=1}$ (for clusters assigned to treatment) and $\boldsymbol{\Omega}^{sorted}_{Z=0}$ (for clusters assigned to control). These two matrices $\boldsymbol{\Omega}^{sorted}_{Z=1}$ and $\boldsymbol{\Omega}^{sorted}_{Z=0}$ can be stacked to yield $\boldsymbol{\Omega}^{sorted}$. This process allows one to carry out a stratified balanced cluster bootstrap, where the cluster-level intervention assignment Z defines the strata.

A further level of stratification by group or group composition may be necessary to ensure that each bootstrap sample contains members of each social group $R = r$, so that intervention effects on disparity can be calculated within each bootstrap sample. For example, while clinical sites in theory will provide care to persons from any social group, they may in practice only provide care for persons from a particular social group (for example, only Black patients may be represented within a practice, because of segregation in residential housing and healthcare access). We observed such a pattern in our motivating example. To address this, one can stratify the clinics jointly according to the intervention arm Z and the pattern of social group presence L (formally, $L = \{I(P(R = 0) > 0), I(P(R = 1) > 0)\}$ where $I(\cdot)$ equals one if its argument is true and equals zero otherwise. Then, steps 1 through 5 are carried out for each set of clusters defined by Z and L to produce the set of matrices $\mathbf{\Omega}^{sorted}_{Z=z,L=l}$ which are stacked to yield $\mathbf{\Omega}^{sorted}$.

Bootstrap Procedure, Estimation, and Hypothesis Testing

To carry out the bootstrap procedure, form $\mathcal{N}$ bootstrap samples $\mathbb{S}_n$ iteratively using the cluster identification numbers specified by columns of the Bootstrap Sampling Matrix $\mathbf{\Omega}^{sorted}$ described in the previous sub-section. For each bootstrap sample $\mathbb{S}_n$, carry out the statistical analysis and store the resulting point estimate $\hat{\theta}_n$ as the nth element in a vector of dimension $1\times \mathcal{N}$ denoted as $\boldsymbol{\psi}^{estimate}$.

On the additive effect scale, the 95% confidence interval for $\hat{\theta}$ is given by the 2.5^{th} and 97.5^{th} percentiles of $\hat{\theta}_n$ within $\boldsymbol{\psi}^{estimate}$. On the relative effect scale, the 95% confidence interval is given by the exponentiating the 2.5^{th} and 97.5^{th} percentiles of $\log(\hat{\theta}_n)$. On the additive effect scale, the standard error of $\hat{\theta}$ (i.e., $s.e.(\hat{\theta})$) can be obtained by taking the standard deviation of $\hat{\theta}$ across $\boldsymbol{\psi}^{estimate}$.

Simulation Procedure for Sample Size Determination

We outline a simulation-based procedure for sample size determination under a desirable level of precision (i.e., confidence interval width). Our approach is adapted from the excellent tutorial by Landau and Stahl (2013), and we refer readers there for additional details, best practices, and examples. The procedure involves specifying a population model, a sampling strategy, an analytic method, and a performance metric. The specified population model and sampling strategies are used to repeatedly simulate sample observed data, to which the analysis metric is applied. Across the estimates obtained for each simulated sample, we may compute the precision (i.e., confidence interval width or average standard error).

Step 1. Population model

Assume a population of sample size $\mathbb{N}_S$ with social group status R (1=marginalized, 0=privileged), one normally distributed allowable covariate A and one binomially distributed non-allowable covariate N. The population is randomly sorted into $\mathbb{N}_H$ clusters H, where each jth cluster is randomly assigned an intervention arm Z (1=treatment, 0=control), and a binary outcome Y for the SATE-D (or D for the SITE-D) is generated as a function of (R, A, N, Z) in the case of Y (or (R, A, N, Z, Y) in the case of D), cluster-level random intercepts, and a binomial error term. The binomial distributions are made covariate-specific by defining the event probability as a logistic regression function. The standard population is the entire sample, so $T = 1$ for everyone.

<u>Observed Data Model</u> (for Sample $\mathbb{S}_{obs}$ of size $\mathbb{N}_S$)

$A \sim N(\mu_A, \sigma_A)$ where $\mu_A = 60$ and $\sigma_A = 12$

$R \sim Bin(p_R)$ where $p_R = (1 + \exp(\beta_0^R + \beta_A^R A)^{-1})^{-1}$, $\beta_0^R = 3.9$, $\beta_A^R = -.06$

$N \sim Bin(p_N)$ where $p_N = (1 + \exp(\beta_0^N + \beta_A^N A + \beta_R^N R)^{-1})^{-1}$, $\beta_0^N = .6$, $\beta_A^N = -.01$, $\beta_R^N = .9$

$H \sim Mult(p_H^1, \dots, P_H^{\mathbb{N}_H})$ where $p_H^j = \mathbb{N}_H / \mathbb{N}_S$ for each jth cluster

$Z_H \sim Bin(p_Z)$ where $p_Z = .5$

$Y \sim Bin(P_Y)$

where $p_Y = \left(1 + \exp\left(\mu_{0,j}^Y(H) + \beta_0^Y + \beta_A^Y A + \beta_R^Y R + \beta_{ZR}^Y Z * R + \beta_N^Y N + \beta_Z^Y Z\right)^{-1}\right)^{-1}$,
$\beta_0^Y = -.5$, $\beta_A^Y = -.0015$, $\beta_R^Y = -.02$, $+\beta_{ZR}^Y = .4$, $\beta_N^Y = .04$, $\beta_Z^Y = -.02$,
$\mu_{0,j}^Y \sim N(0, \sigma_{0,j}^Y)$ e.g., where $\sigma_{0,j}^Y = .0025$

$D \sim Bin(P_D)$

where $P_D = \left(1 + \exp\left(\mu_{0,j}^D(H) + \beta_0^D + \beta_A^D A + \beta_R^D R + \beta_{ZR}^D Z * R + \beta_N^D N + \beta_Z^D Z + \beta_Y^D Y\right)^{-1}\right)^{-1}$,
$\beta_0^D = -.9$, $\beta_A^D = -.01$, $\beta_R^D = -.5$, $\beta_N^D = .02$, $\beta_Z^D = -.2$, $\beta_Y^D = .7$
$\mu_{0,j}^D \sim N(0, \sigma_{0,j}^D)$ e.g., where $\sigma_{0,j}^D = .0012$

$T = 1$ with probability one

(If the marginalized group is chosen as the standard population, then we set $T = R$).

<u>Counterfactual Data Model under Intervention to Set Z to Value z^* among Social Group $R = r^*$</u> (for a Population $\mathbb{P}_{cnt}^{(z^*,r^*)}$ of size $\mathbb{N}_S * \mathbb{N}_{big}$); $\mathbb{N}_{big}$ is a very, very large number e.g. 10^8).

The variables (R, A, N, H, Y, D, T) for the SATE-D (or (R, A, N, H, D, T)) are generated as in the observed data model. However, in the Counterfactual Data Model, we make a few modifications:

$R = r^*$ with probability one, where r^* is 1 when simulating the marginalized group, 0 otherwise.

$Z = z^*$ with probability one, where z^* is 1 when simulating all clusters treated, 0 otherwise.

Additionally, for the SITE-D, we make an additional modification:

$Y \sim P(Y^{z^*} = 1 | T = 1)$ where Y^{z^*} is the draw under setting Z to z^*.

Under the SITE-D, if $T = 1$ excludes those with $R = r^*$, this is obtained separately for the Counterfactual Data Model under $T = 1$. For example, if $R = 1$ implies $T = 1$, $P(Y^{z^*} = 1 | T = 1)$ is obtained under $r^* = 1$.

For either the SATE-D or SITE-D, the assignment of R achieves the balancing of the allowable A such that $\mathbb{P}_{cnt}^{(z^*,1)}$ and $\mathbb{P}_{cnt}^{(z^*,0)}$ have the same distribution of A (to meaningfully define disparity).

Note also that if the marginalized group is chosen as the standard population, then we modify the data generating model for the counterfactual population as follows. First, after generating A and R for $\mathbb{N}_S * \mathbb{N}_{big}$ observations, we restrict the vector A to the $\mathbb{N}_S^\dagger * \mathbb{N}_{big}$ observations where $R = 1$. $\mathbb{N}_S^\dagger$ is the sample-specific number of observations where $R = 1$. We then continue to generate (N, H, Y, D, T) for these $\mathbb{N}_S^\dagger * \mathbb{N}_{big}$ observations as described previously. The final dataset will have $\mathbb{N}_S^\dagger * \mathbb{N}_{big}$ observations.

Step 2: Sampling Strategy

To generate a sample of the observed data $\mathbb{S}_{obs}$, repeat its data generating process $\mathbb{N}_S$ times. To generate counterfactual population data $\mathbb{P}_{cnt}^{(z^*,r^*)}$ under a given choice of (z^*, r^*), repeat its generating data process $\mathbb{N}_S * \mathbb{N}_{big}$ times, where $\mathbb{N}_{big}$ is as large as computationally feasible.

Step 3. Analytic method.

For the SATE-D, the true value for each $\tilde{\mu}^{z^*}(r^*)$ can be obtained by taking the mean of Y in each generated dataset $\mathbb{S}_{cnt}^{(z^*,r^*)}$. Thus, taking such means from the generated data $\mathbb{P}_{cnt}^{(1,1)}$, $\mathbb{P}_{cnt}^{(0,1)}$, $\mathbb{P}_{cnt}^{(1,0)}$, and $\mathbb{P}_{cnt}^{(0,0)}$ provides the true values for $\tilde{\mu}^1(1)$, $\tilde{\mu}^0(1)$, $\tilde{\mu}^1(0)$, $\tilde{\mu}^0(0)$, with which the true intervention effect can be calculated according the equation (6) in the main text.

For the SATE-D, the estimated value for each $\tilde{\mu}^{z^*}(r^*)$ can be obtained applying either the weighting estimator $\pi(x)$ (see equation (7) of the main text) or the g-computation estimator $\delta(x)$ (see equation (9) of the main text) to the generated sample $\mathbb{S}_{obs}$. Their 95% confidence intervals, standard errors, and p-values of the null hypothesis of no intervention effect are obtained by

applying the bootstrap procedure described in the previous section to each of the generated samples $\mathbb{S}_{obs}$. A similar approach is used to obtain the true and estimated values for the SITE-D.

Step 4. Performance.

We are ready to compute the precision after we chosen a design and reflected it in the population model of sample size $\mathbb{N}_s$ (Step 1), generated a large number (e.g., 1,000) of samples of the observed data $\mathbb{S}_{obs}$ (Step 2), and applied a chosen estimator, weighting $\pi(x)$ or g-computation $\delta(x)$, and bootstrapping procedure to each of the samples and recorded the standard error (to estimate precision). Given completion of these steps, we can compute the precision by the average standard error of the estimator, $s.e.(\hat{\theta})$ among the samples $\mathbb{S}_{obs}$, or as the average width of the 95% confidence intervals among the samples $\mathbb{S}_{obs}$. Landau and Stahl (2013) describe additional metrics such as the type-I-error rate and the confidence interval coverage. Repeating this process across a range of sample sizes $\mathbb{N}_s$ and determining their precision (and plotting $\mathbb{N}_s$ versus precision) allows us to determine a sample size with a desirable level of precision.

Simulation Study of Proposed Estimators

We carried out a simulation study to briefly evaluate the validity of the estimation procedures (weighting and g-computation) and inference procedures (the balanced stratified cluster bootstrap) procedure described in the main text. We generated the counterfactual and observed data sets as described in the previous section (Simulation Procedure for Sample Size Determination) with the standard population set to be the marginalized group, such that $T = R$. The counterfactual population size was set to be $2\text{x}10^9$ and observed sample size was set to be 1800, each with 30 clusters. After simulating and recording the estimates of the true values of the SATE-D and SITE-D intervention effect, we carried out 1,000 simulations for the weighting and g-computation estimators proprosed for these estimands. We calculated the following performance metrics: bias, root mean square error (RMSE), standard error (S.E.), and confidence interval coverage. The results are shown in sTable 4 below. We find that for both the SATE-D (true value = .099217) and the SITE-D (true value = -0.00695), the proposed weighting and g-computation estimators for these estimands are consistent. However, the proposed bootstrap procedure has coverage slightly below the advertised nominal rate of 95%.

sTable 4. Statistical Performance of Weighting and G-computation Estimators for SATE-D and SITE-D

	Bias	RMSE	S.E.	Coverage (%)
SATE-D				
Weighting	0.0004	0.0530	0.0513	92.9
g-computation	-0.0006	0.0528	0.0500	92.5
SITE-D				
Weighting	0.0070	0.0472	0.0454	92.8
g-computation	-0.0028	0.0455	0.0452	92.9

Proof of Results

Notation

Let the symbol $V \coprod W | X$ represent statistical independence between V and W given X. Let the expression $P(x|v)$ stand as shorthand for the probability statement $P(X = x|V = v)$, and the expression $E[X|v)$ stand as shorthand for $E[X|V = v]$. Denote the social group $R \in (0,1)$, the standard population $T \in (0,1)$ where t^* is the value chosen to represent membership in the standard population (in the main text the value is chosen as '1'), intervention $Z \in (0,1)$ where z^* is the value chosen to represent treatment, C as loss to follow-up $C \in (0,1)$ where c^* is the value chosen to represent retention in the study at follow-up (typically the value is chosen as '0'), Y as the outcome measurement for a health status outcome, D is the outcome measurement for a decision outcome. Let $\boldsymbol{A}$ represent the partition of observed baseline covariates that are deemed as allowable and let $\boldsymbol{N}$ represent an additional set of auxiliary non-allowable variables within the remaining set of baseline covariates that are used for further adjustment (e.g., for confounding). $\boldsymbol{B}$ are the post-intervention criteria relevant to the decision outcome D.

Formal Statement of Assumptions

<u>Assumptions for Total Effects (SATE-D) without Study Attrition</u>

A1) $P(r|z,\boldsymbol{a}) > 0$ for all $\boldsymbol{A}$ with $P(\boldsymbol{a}|t^*) > 0$, i.e., overlap of $\boldsymbol{A}$ w.r.t. R and T

A2) $Y^z \coprod Z|R,\boldsymbol{N},\boldsymbol{A}$ for all z, i.e., conditional exchangeability w.r.t. Z

A3) $P(z|r,\boldsymbol{n},\boldsymbol{a}) > 0$ for all $(\boldsymbol{N},\boldsymbol{A})$ with $P(\boldsymbol{n},\boldsymbol{a}|r) > 0$, i.e., posititivity w.r.t. Z

A4) If $Z = z$ then $Y^z \equiv Y$, i.e., consistency w.r.t. Z

<u>Assumptions for Total Effects (SATE-D) with Study Attrition</u>

A1 (see above)

B1) $Y^{(z,c^*)} \coprod C,Z|R,\boldsymbol{N},\boldsymbol{A}$ for all z, i.e., conditional exchangeability w.r.t. $(C = c^*, Z)$

B2) $P(c^*,z|r,\boldsymbol{n},\boldsymbol{a}) > 0$ for all $(\boldsymbol{N},\boldsymbol{A})$ with $P(\boldsymbol{n},\boldsymbol{a}|r) > 0$, i.e., positivity w.r.t. $(C = c^*, Z)$

B3) If $Z = z$ and $C = c^*$ then $Y^{(z,c^*)} \equiv Y$, i.e., consistency w.r.t. $(C = c^*, Z)$

<u>Assumptions for Direct Effects (SITE-D) without Study Attrition</u>

A1 (see above), and A2, A3, and A4 (see above, with respect to $D^{(z,\boldsymbol{G}=\boldsymbol{b})}$ rather than Y^z)

C1) $P(r|z,\boldsymbol{n},\boldsymbol{a},\boldsymbol{b}) > 0$ for all $\boldsymbol{B}$ with $P(\boldsymbol{b}|z,t^*,\boldsymbol{n},\boldsymbol{a}) > 0$, i.e., overlap of $\boldsymbol{B}$ w.r.t. R and T where $P(\boldsymbol{b}|z,t^*,\boldsymbol{n},\boldsymbol{a}) = P(\boldsymbol{B}^Z = \boldsymbol{b}|t^*,\boldsymbol{n},\boldsymbol{a})$ under C2, A3, and C4

C2) $D^{(z,G=\boldsymbol{b})} \coprod \boldsymbol{B}|Z,R,\boldsymbol{N},\boldsymbol{A}$ and $\boldsymbol{B}^{\boldsymbol{z}} \coprod Z|T = t^*,\boldsymbol{N},\boldsymbol{A}$ for all z

C3) $P(\boldsymbol{b}|z,r,\boldsymbol{n},\boldsymbol{a}) > 0$ for all $(\boldsymbol{N},\boldsymbol{A})$ with $P(\boldsymbol{n},\boldsymbol{a}|z,r) > 0$ and all $\boldsymbol{B}$ with $P(\boldsymbol{b}|z,t^*,\boldsymbol{n},\boldsymbol{a}) > 0$, and all z, i.e., positivity w.r.t. $\boldsymbol{B}$

C4) If $Z = z$ then $\boldsymbol{B}^z \equiv \boldsymbol{B}$. If also $\boldsymbol{B} = \boldsymbol{b}$ then $D^{(z,G=\boldsymbol{b})} \equiv D$, i.e., consistency w.r.t. $(\boldsymbol{B}, Z)$

<u>Assumptions for Direct Effects (SITE-D) with Study Attrition</u>

A1 (see above), and B1, B2, and B3 (see above, with respect to $D^{(z,c^*,\boldsymbol{G}=\boldsymbol{b})}$ rather than $Y^{(z,c^*)}$)

D1) $P(r|c^*,z,\boldsymbol{n},\boldsymbol{a},\boldsymbol{b}) > 0$ for all $\boldsymbol{B}$ with $P(\boldsymbol{b}|c^*,z,t^*,\boldsymbol{n},\boldsymbol{a}) > 0$, i.e., overlap of $\boldsymbol{B}$ w.r.t. R and T where $P(\boldsymbol{b}|c^*,z,t^*,\boldsymbol{n},\boldsymbol{a}) = P(\boldsymbol{B}^{(z,c^*)} = \boldsymbol{b}|t^*,\boldsymbol{n},\boldsymbol{a})$ under D2, B2, and D4

D2) $D^{(z,c^*,G=\boldsymbol{b})} \coprod \boldsymbol{B}|C = c^*,Z,R,\boldsymbol{N},\boldsymbol{A}$ and $\boldsymbol{B}^{(z,c^*)} \coprod (C,Z)|T = t^*,\boldsymbol{N},\boldsymbol{A}$ for all z, i.e., cond. exch.

D3) $P(\boldsymbol{b}|c^*,z,r,\boldsymbol{n},\boldsymbol{a}) > 0$ for all $(\boldsymbol{N},\boldsymbol{A})$ with $P(\boldsymbol{n},\boldsymbol{a}|c^*,z,r) > 0$, and all $\boldsymbol{B}$ with $P(\boldsymbol{b}|c^*,z,t^*,\boldsymbol{n},\boldsymbol{a}) > 0$ and all z, i.e., positivity w.r.t. $\boldsymbol{B}$

D4) If $Z = z$ and $C = c^*$ then $\boldsymbol{B}^{(z,c^*)} \equiv \boldsymbol{B}$. If also $\boldsymbol{B} = \boldsymbol{b}$ then $D^{(z,c^*,G=\boldsymbol{b})} \equiv D$, i.e., consistency w.r.t. $(\boldsymbol{B}, C, Z)$

Assumptions for Total Effects (SATT-D) without Study Attrition

A1*) $P(r|z,\boldsymbol{a}) > 0$ for all $\boldsymbol{A}$ with $P(\boldsymbol{a}|t^*,z^*) > 0$, i.e., overlap of $\boldsymbol{A}$ w.r.t. R and T

A2*) $Y^{Z\neq z^*} \coprod Z|R,\boldsymbol{N},\boldsymbol{A}$ i.e., conditional exchangeability w.r.t. $Z \neq z^*$

A3*) $P(Z \neq z^*|r,\boldsymbol{n},\boldsymbol{a}) > 0$ for all $(\boldsymbol{N},\boldsymbol{A})$ with $P(\boldsymbol{n},\boldsymbol{a}|r,z^*) > 0$, i.e., posititivity w.r.t. $Z \neq z^*$

A4*) If $Z = z$ then $Y^z \equiv Y$, i.e., consistency w.r.t. Z

Assumptions for Total Effects (SATT-D) with Study Attrition

A1* (see above)

B1*) $Y^{(Z\neq z^*,c^*)} \coprod C,Z|R,\boldsymbol{N},\boldsymbol{A}$ and $Y^{(Z=z^*,c^*)} \coprod C|Z = z^*,R,\boldsymbol{N},\boldsymbol{A}$, i.e., cond. exch. w.r.t. $(C = c^*, Z \neq z^*)$ and $(C = c^*, Z = z^*)$

B2*) $P(c^*, Z \neq z^*|r,\boldsymbol{n},\boldsymbol{a}) > 0$ and $P(c^*|z^*,r,\boldsymbol{n},\boldsymbol{a}) > 0$ for all $(\boldsymbol{N},\boldsymbol{A})$ with $P(\boldsymbol{n},\boldsymbol{a}|r,z^*) > 0$, i.e., positivity w.r.t. $(C = c^*, Z \neq z^*)$ and $(C = c^*, Z = z^*)$

B3*) If $Z = z$ and $C = c^*$ then $Y^{(z,c^*)} \equiv Y$, i.e., consistency w.r.t. $(C = c^*, Z)$

Assumptions for Direct Effects (SITT-D) without Study Attrition

A1* (see above), A2*, A3*, and A4* (see above, with respect to $D^{(Z\neq z^*,\boldsymbol{G}=\boldsymbol{b})}$ rather than Y^z)

C1*) $P(r|z^*,\boldsymbol{n},\boldsymbol{a},\boldsymbol{b}) > 0$ for all $\boldsymbol{B}$ with $P(\boldsymbol{b}|z^*,t^*,\boldsymbol{n},\boldsymbol{a}) > 0$, i.e., overlap of $\boldsymbol{B}$ w.r.t. R and T where $P(\boldsymbol{b}|z^*,t^*,\boldsymbol{n},\boldsymbol{a}) = P(\boldsymbol{B}^z = \boldsymbol{b}|z^*,t^*,\boldsymbol{n},\boldsymbol{a})$ under C2*, A3*, and C4*

C2*) $D^{(z,G=\boldsymbol{b})} \coprod \boldsymbol{B}|Z,R,\boldsymbol{N},\boldsymbol{A}$ and $\boldsymbol{B}^{Z\neq z^*} \coprod Z|T = t^*,\boldsymbol{N},\boldsymbol{A}$, i.e. conditional exch. w.r.t. $\boldsymbol{B}$

C3*) $P(\boldsymbol{b}|z,r,\boldsymbol{n},\boldsymbol{a}) > 0$ for all $(\boldsymbol{N},\boldsymbol{A})$ with $P(\boldsymbol{n},\boldsymbol{a}|z^*,r) > 0$ and all $\boldsymbol{B}$ with $P(\boldsymbol{b}|z^*,t^*,\boldsymbol{n},\boldsymbol{a}) >$ 0, and all z, i.e., positivity w.r.t. $\boldsymbol{B}$

C4*) If $Z = z$ then $\boldsymbol{B}^z \equiv \boldsymbol{B}$. If also $\boldsymbol{B} = \boldsymbol{b}$ then $D^{(z,G=\boldsymbol{b})} \equiv D$, i.e., consistency w.r.t. $(\boldsymbol{B}, Z)$

Assumptions for Direct Effects (SITT-D) with Study Attrition

A1* (see above), B1*, B2*, and B3* (see above, with respect to $D^{(z,c^*,\boldsymbol{G}=\boldsymbol{b})}$ rather than $Y^{(z,c^*)}$)

D1*) $P(r|c^*,z^*,\boldsymbol{n},\boldsymbol{a},\boldsymbol{b}) > 0$ for all $\boldsymbol{B}$ with $P(\boldsymbol{b}|c^*,z^*,t^*,\boldsymbol{n},\boldsymbol{a}) > 0$, i.e., overlap of $\boldsymbol{B}$ w.r.t. R and T where $P(\boldsymbol{b}|c^*,z^*,t^*,\boldsymbol{n},\boldsymbol{a}) = P(\boldsymbol{B}^{(z,c^*)} = \boldsymbol{b}|z^*,t^*,\boldsymbol{n},\boldsymbol{a})$ under D2*, B2*, and D4*

D2*) $D^{z,c^*,G=\boldsymbol{b}} \coprod \boldsymbol{B}|C = c^*,Z,R,\boldsymbol{N},\boldsymbol{A}$ and $\boldsymbol{B}^{(Z\neq z^*,c^*)} \coprod (C,Z)|T = t^*,\boldsymbol{N},\boldsymbol{A}$ and $\boldsymbol{B}^{(Z=z^*,c^*)} \coprod C|Z = z^*,T = t^*,\boldsymbol{N},\boldsymbol{A}$

D3*) $P(\boldsymbol{b}|c^*,z,r,\boldsymbol{n},\boldsymbol{a}) > 0$ for all $(\boldsymbol{N},\boldsymbol{A})$ with $P(\boldsymbol{n},\boldsymbol{a}|c^*,z^*,r) > 0$, and all $\boldsymbol{B}$ with $P(\boldsymbol{b}|c^*,z^*,t^*,\boldsymbol{n},\boldsymbol{a}) > 0$ and all z, i.e., positivity w.r.t. $\boldsymbol{B}$,

D4*) If $Z = z$ and $C = c^*$ then $\boldsymbol{B}^{(z,c^*)} \equiv \boldsymbol{B}$. If also $\boldsymbol{B} = \boldsymbol{b}$ then $D^{(z,c^*,G=\boldsymbol{b})} \equiv D$, i.e., consistency w.r.t. $(\boldsymbol{B}, C, Z)$

Proofs

<u>Sample Average Effect of Treatment on Disparity (SATE-D)</u>

Estimand

$$\tilde{\mu}^{z}(r) = \sum_{\boldsymbol{a}} E\left[Y^{(z,c^*)}\middle|r, \boldsymbol{a}\right]P(\boldsymbol{a}|t^*) \tag{14}$$

Identifying Formula for (14)

$$\begin{aligned}
&\sum_{\boldsymbol{a}} E\left[Y^{(z,c^*)}\middle|r, \boldsymbol{a}\right]P(\boldsymbol{a}|t^*) \\
&= \sum_{\boldsymbol{a},\boldsymbol{n}} E\left[Y^{(z,c^*)}\middle|r, \boldsymbol{n}, \boldsymbol{a}\right]P(\boldsymbol{n}|r, \boldsymbol{a})P(\boldsymbol{a}|t^*) \\
&= \sum_{\boldsymbol{a},\boldsymbol{n}} E\left[Y^{(z,c^*)}\middle|c^*, z, r, \boldsymbol{n}, \boldsymbol{a}\right]P(\boldsymbol{n}|r, \boldsymbol{a})P(\boldsymbol{a}|t^*) \\
&= \sum_{\boldsymbol{a},\boldsymbol{n}} E[Y|c^*, z, r, \boldsymbol{n}, \boldsymbol{a}]P(\boldsymbol{n}|r, \boldsymbol{a})P(\boldsymbol{a}|t^*)
\end{aligned} \tag{15}$$

The expression on the LHS of the first line is the estimand and holds by the overlap assumption A1, the first equality holds by the total law of probability, the second by conditional exchangeability A2 (and B1 under study attrition) and positivity A3 (and B2 under study attrition), and the third by consistency A4 (and B3 under study attrition). Note that for the SATE-D the standard population $T = t^*$ is chosen among the entire sample $Z = z^* \cup Z \neq z^*$.

Weighting Estimator for (14)

$$\begin{aligned}
&\sum_{\boldsymbol{a},\boldsymbol{n}} E[Y|c^*, z, r, \boldsymbol{n}, \boldsymbol{a}]P(\boldsymbol{n}|r, \boldsymbol{a})P(\boldsymbol{a}|t^*) \\
&= \sum_{\boldsymbol{a},\boldsymbol{n}} E[Y|c^*, z, r, a]P(\boldsymbol{n}|c^*, z, r, \boldsymbol{a})P(\boldsymbol{a}|c^*, r, z)\frac{P(\boldsymbol{n}|r, \boldsymbol{a})}{P(\boldsymbol{n}|c^*, z, r, \boldsymbol{a})}\frac{P(\boldsymbol{a}|t^*)}{(\boldsymbol{a}|c^*, r, z)} \\
&= \sum_{\boldsymbol{a},\boldsymbol{n}} \begin{pmatrix} E[Y|c^*, z, r, a]P(\boldsymbol{n}|c^*, z, r, \boldsymbol{a})P(\boldsymbol{a}|c^*, r, z) \\ \times \frac{P(c^*|z, r)}{P(c^*|z, r, \boldsymbol{n}, \boldsymbol{a})}\frac{P(Z|r)}{P(Z|r, \boldsymbol{n}, \boldsymbol{a})}\frac{P(t^*|\boldsymbol{a})}{P(r|\boldsymbol{a})}\frac{P(r)}{P(t^*)} \end{pmatrix} \\
&= E[W_{r,z}^{SATE-D} \times W_c \times Y|c^*, z, r]
\end{aligned}$$

$$\text{where } W_{r,z}^{SATE-D} = \frac{P(Z|r)}{P(Z|r, \boldsymbol{n}, \boldsymbol{a})}\frac{P(t^*|a)}{P(r|a)}\frac{P(r)}{P(t^*)} \text{ and } W_c = \frac{P(c^*|z,r)}{P(c^*|z,r,\boldsymbol{n},\boldsymbol{a})} \tag{16}$$

The second equality holds by bayes rule and simplification.

G-computation Estimator for (14)

$$\begin{aligned}
&\sum_{\boldsymbol{a},\boldsymbol{n}} E[Y|c^*, z, r, \boldsymbol{n}, \boldsymbol{a}]P(\boldsymbol{n}|r, \boldsymbol{a})P(\boldsymbol{a}|t^*) \\
&= \sum_{\boldsymbol{a}} E(E[Y|c^*, z, r, \boldsymbol{n}, \boldsymbol{a}]|r, \boldsymbol{a})P(\boldsymbol{a}|t^*) \\
&= E⟦E(E[Y|c^*, z, r, \boldsymbol{n}, \boldsymbol{a}]|r, \boldsymbol{a})|t^*⟧
\end{aligned}$$

$$\text{where } E(\cdot) \text{ is over } P(\boldsymbol{n}|r, \boldsymbol{a}) \text{ and } E⟦\cdot⟧ \text{ is over } P(\boldsymbol{a}|t^*) \tag{17}$$

The equalities holds by definition of an iterated conditional expectation.

Sample Average Effect of Treatment on Disparity Among the Treated (SATT-D)

Estimand

$$\tilde{\mu}^{z}(r) = \sum_{\boldsymbol{a}} E\left[Y^{(z,c^*)}\middle|z^*, r, \boldsymbol{a}\right] P(\boldsymbol{a}|z^*, t^*) \tag{18}$$

Identifying Formula for (18)

$$\begin{aligned} &\sum_{\boldsymbol{a}} E\left[Y^{(z,c^*)}\middle|z^*, r, \boldsymbol{a}\right] P(\boldsymbol{a}|z^*, t^*) \\ &= \sum_{\boldsymbol{a},\boldsymbol{n}} E\left[Y^{(z,c^*)}\middle|z^*, r, \boldsymbol{n}, \boldsymbol{a}\right] P(\boldsymbol{n}|z^*, r, \boldsymbol{a}) P(\boldsymbol{a}|z^*, t^*) \\ &= \sum_{\boldsymbol{a},\boldsymbol{n}} E\left[Y^{(z,c^*)}\middle|c^*, z, r, \boldsymbol{n}, \boldsymbol{a}\right] P(\boldsymbol{n}|z^*, r, \boldsymbol{a}) P(\boldsymbol{a}|z^*, t^*) \\ &= \sum_{\boldsymbol{a},\boldsymbol{n}} E[Y|c^*, z, r, \boldsymbol{n}, \boldsymbol{a}] P(\boldsymbol{n}|z^*, r, \boldsymbol{a}) P(\boldsymbol{a}|z^*, t^*) \end{aligned} \tag{19}$$

The expression on the LHS of the first line is the estimand and holds by the overlap assumption A1*, the first equality holds by the total law of probability, the second by conditional exchangeability A2* (and B1* under study attrition), and positivity A3* (and B2* under study attrition), and the third by consistency A4* (and B3* under study attrition). Note that for the SATT-D the standard population t^* is chosen among the treated $Z = z^*$.

Weighting Estimator for (18)

$$\begin{aligned} &\sum_{a,\boldsymbol{n}} E[Y|c^*, z, r, \boldsymbol{n}, \boldsymbol{a}] P(\boldsymbol{n}|z^* r, \boldsymbol{a}) P(\boldsymbol{a}|z^*, t^*) \\ &= \sum_{\boldsymbol{a},\boldsymbol{n}} E[Y|c^*, z, r, a] P(\boldsymbol{n}|c^*, z, r, \boldsymbol{a}) P(\boldsymbol{a}|c^*, z, r) \frac{P(\boldsymbol{n}|z^*, r, \boldsymbol{a})}{P(\boldsymbol{n}|c^*, z, r, \boldsymbol{a})} \frac{P(\boldsymbol{a}|z^*, t^*)}{(\boldsymbol{a}|c^*, z, r)} \\ &= \sum_{\boldsymbol{a},\boldsymbol{n}} \begin{pmatrix} E[Y|c^*, z, r, a] P(\boldsymbol{n}|c^*, z, r, \boldsymbol{a}) P(\boldsymbol{a}|c^*, r, z) \\ \times \frac{P(c^*|z, r)}{P(c^*|z, r, \boldsymbol{n}, \boldsymbol{a})} \frac{P(z^*|r, \boldsymbol{n}, \boldsymbol{a})}{P(Z|r, \boldsymbol{n}, \boldsymbol{a})} \frac{P(t^*, z^*|\boldsymbol{a})}{P(r, z^*|\boldsymbol{a})} \frac{P(r,z)}{P(t^*,z^*)} \end{pmatrix} \\ &= E[W_{r,z}^{SATT-D} \times W_c \times Y|c^*, z, r] \\ &\quad \text{where } W_{r,z}^{SATT-D} = \frac{P(z^*|r, \boldsymbol{n}, \boldsymbol{a})}{P(Z|r, \boldsymbol{n}, \boldsymbol{a})} \frac{P(t^*, z^*|\boldsymbol{a})}{P(r, z^*|\boldsymbol{a})} \frac{P(r,z)}{P(t^*,z^*)} \text{ and } W_c = \frac{P(c^*|z,r)}{P(c^*|z,r,\boldsymbol{n},\boldsymbol{a})} \end{aligned} \tag{20}$$

The second equality bayes rule and simplification. Note that the estimation of the expression $\sum_{\boldsymbol{a},\boldsymbol{n}} E[Y|c^*, z^*, r, \boldsymbol{n}, \boldsymbol{a}] P(\boldsymbol{n}|z^* r, \boldsymbol{a}) P(\boldsymbol{a}|z^*, t^*)$ does not require the first term of $W_{r,z}^{SATT-D}$.

G-computation Estimator for (18)

$$\begin{aligned} &\sum_{\boldsymbol{a},\boldsymbol{n}} E[Y|c^*, z, r, \boldsymbol{n}, \boldsymbol{a}] P(\boldsymbol{n}|z^*, r, \boldsymbol{a}) P(\boldsymbol{a}|z^*, t^*) \\ &= \sum_{\boldsymbol{a}} E(E[Y|c^*, z, r, \boldsymbol{n}, \boldsymbol{a}]|z^*, r, \boldsymbol{a}) P(\boldsymbol{a}|z^*, t^*) \\ &= E[\![E(E[Y|c^*, z, r, \boldsymbol{n}, \boldsymbol{a}]|z^*, r, \boldsymbol{a})|z^*, t^*]\!] \\ &\quad \text{where } E(\cdot) \text{ is over } P(\boldsymbol{n}|z^*, r, \boldsymbol{a}) \text{ and } E[\![\cdot]\!] \text{ is over } P(\boldsymbol{a}|z^*, t^*) \end{aligned} \tag{21}$$

The equality holds by definition of an iterated conditional expectation.

Sample Interventional Direct Effect of Treatment on Disparity (SITE-D)

Estimand

$$
\begin{aligned}
&\ddot{\mu}^{z}(r)\\
&=\textstyle\sum_{\boldsymbol{a}} E\left[D^{(z,c^*,\boldsymbol{G}=\boldsymbol{b})}\middle|r,\boldsymbol{a}\right]P(\boldsymbol{a}|t^*)\\
&=\textstyle\sum_{\boldsymbol{a},\boldsymbol{b}} E\left[D^{(z,c^*,\boldsymbol{G}=\boldsymbol{b})}\middle|\boldsymbol{G}=\boldsymbol{b},r,\boldsymbol{a}\right]P(\boldsymbol{G}=\boldsymbol{b}|r,\boldsymbol{a})P(\boldsymbol{a}|t^*) \qquad (22)
\end{aligned}
$$

Identifying Formula for (22)

$$
\begin{aligned}
&\textstyle\sum_{\boldsymbol{a},\boldsymbol{b}} E\left[D^{(z,c^*,\boldsymbol{G}=\boldsymbol{b})}\middle|\boldsymbol{G}=\boldsymbol{b},r,\boldsymbol{a}\right]P(\boldsymbol{G}=\boldsymbol{b}|r,\boldsymbol{a})P(\boldsymbol{a}|t^*)\\
&=\textstyle\sum_{\boldsymbol{a},\boldsymbol{b}} E\left[D^{(z,c^*,\boldsymbol{b})}\middle|r,\boldsymbol{a}\right]P(\boldsymbol{G}=\boldsymbol{b}|r,\boldsymbol{a})P(\boldsymbol{a}|t^*)\\
&=\textstyle\sum_{\boldsymbol{a},\boldsymbol{b},\boldsymbol{n}} E\left[D^{(z,c^*,\boldsymbol{b})}\middle|r,\boldsymbol{n},\boldsymbol{a}\right]P(\boldsymbol{G}=\boldsymbol{b}|r,\boldsymbol{n},\boldsymbol{a})P(\boldsymbol{n}|r,\boldsymbol{a})P(\boldsymbol{a}|t^*)\\
&=\textstyle\sum_{\boldsymbol{a},\boldsymbol{b},\boldsymbol{n}} E\left[D^{(z,c^*,\boldsymbol{b})}\middle|r,\boldsymbol{n},\boldsymbol{a}\right]P(\boldsymbol{G}=\boldsymbol{b}|r,\boldsymbol{a})P(\boldsymbol{n}|r,\boldsymbol{a})P(\boldsymbol{a}|t^*)\\
&=\textstyle\sum_{\boldsymbol{a},\boldsymbol{b},\boldsymbol{n}} E\left[D^{(z,c^*,\boldsymbol{b})}\middle|c^*,z,r,\boldsymbol{n},\boldsymbol{a}\right]P(\boldsymbol{G}=\boldsymbol{b}|r,\boldsymbol{a})P(\boldsymbol{n}|r,\boldsymbol{a})P(\boldsymbol{a}|t^*)\\
&=\textstyle\sum_{\boldsymbol{a},\boldsymbol{b},\boldsymbol{n}} E\left[D^{(z,c^*,\boldsymbol{b})}\middle|\boldsymbol{b},c^*,z,r,\boldsymbol{n},\boldsymbol{a}\right]P(\boldsymbol{G}=\boldsymbol{b}|r,\boldsymbol{a})P(\boldsymbol{n}|r,\boldsymbol{a})P(\boldsymbol{a}|t^*)\\
&=\textstyle\sum_{\boldsymbol{a},\boldsymbol{b},\boldsymbol{n}} E[D|\boldsymbol{b},c^*,z,r,\boldsymbol{n},\boldsymbol{a}]P(\boldsymbol{G}=\boldsymbol{b}|r,\boldsymbol{a})P(\boldsymbol{n}|r,\boldsymbol{a})P(\boldsymbol{a}|t^*)\\
&=\textstyle\sum_{\boldsymbol{a},\boldsymbol{b},\boldsymbol{n}} E[D|\boldsymbol{b},c^*,z,r,\boldsymbol{n},\boldsymbol{a}]P\left(B^{(z,c^*)}=\boldsymbol{b}\middle|t^*\right)P(\boldsymbol{n}|r,\boldsymbol{a})P(\boldsymbol{a}|t^*)\\
&=\textstyle\sum_{\boldsymbol{a},\boldsymbol{b},\boldsymbol{n}} \begin{pmatrix} E[D|\boldsymbol{b},c^*,z,r,\boldsymbol{n},\boldsymbol{a}] \\ \times \sum_{\boldsymbol{n},\boldsymbol{a}}\left\{P\left(\boldsymbol{B}^{(z,c^*)}=\boldsymbol{b}\middle|t^*,\boldsymbol{n},\boldsymbol{a}\right)P(\boldsymbol{n},\boldsymbol{a}|t^*)\right\} \\ \times P(\boldsymbol{n}|r,\boldsymbol{a})P(\boldsymbol{a}|t^*) \end{pmatrix}\\
&=\textstyle\sum_{\boldsymbol{a},\boldsymbol{b},\boldsymbol{n}} \begin{pmatrix} E[D|\boldsymbol{b},c^*,z,r,\boldsymbol{n},\boldsymbol{a}] \\ \times \sum_{\boldsymbol{n}}\left\{P\left(\boldsymbol{B}^{(z,c^*)}=\boldsymbol{b}\middle|c^*,z,t^*,\boldsymbol{n},\boldsymbol{a}\right)P(\boldsymbol{n},\boldsymbol{a}|t^*)\right\} \\ \times P(\boldsymbol{n}|r,\boldsymbol{a})P(\boldsymbol{a}|t^*) \end{pmatrix}\\
&=\textstyle\sum_{\boldsymbol{a},\boldsymbol{b},\boldsymbol{n}} E[D|\boldsymbol{b},c^*,z,r,\boldsymbol{n},\boldsymbol{a}]\sum_{\boldsymbol{n},\boldsymbol{a}}\{P(\boldsymbol{b}|c^*,z,t^*,\boldsymbol{n},\boldsymbol{a})P(\boldsymbol{n},\boldsymbol{a}|t^*)\}\,P(\boldsymbol{n}|r,\boldsymbol{a})P(\boldsymbol{a}|t^*) \qquad (23)
\end{aligned}
$$

The expression on the LHS of the first line is the estimand and holds by the overlap assumptions A1, C1 (D1 under study attrition), and the total law of probability. (Overlap C1 (D1 under study attrition) itself requires assumptions C2, A3, and C4 [D2, B2, and D4 under study attrition]). The first equality holds by definition of the intervention $\boldsymbol{G}$ as random given $\boldsymbol{R}=\boldsymbol{r}$ and $\boldsymbol{A}=\boldsymbol{a}$, the second by the total law of probability, the third by definition of the intervention $\boldsymbol{G}$ as a random assignment, the fourth by conditional exchangeability A2 (and B1 under study attrition), and positivity A3 (and B2 under study attrition), and the fifth by conditional exchangeability C2 (D2 under study attrition) and positivity C3 (D3) under study attrition, the sixth by consistency C4 (D4 under study attrition), the seventh by definition of the intervention $\boldsymbol{G}$ as a random draw from the conditional counterfactual distribution of $\boldsymbol{B}$ under assignment of Z to value z among the standard population $T=t^*$, the eighth by the total law of probability, the ninth by conditional exchangeability C2 (D2 under study attrition) and positivity A3 (and B2 under study attrition), and the tenth by consistency C4 (D4 under study attrition). For the SITE-D the standard population $T=t^*$ is chosen among the entire sample $Z=z^*\cup Z\neq z^*$.

Proof of Weighting Estimator for (22)

$$
\begin{aligned}
&\textstyle\sum_{\boldsymbol{a},\boldsymbol{b},\boldsymbol{n}} E[D|\boldsymbol{b},c^*,z,r,\boldsymbol{n},\boldsymbol{a}] \sum_{\boldsymbol{n},\boldsymbol{a}}\{P(\boldsymbol{b}|c^*,z,t^*,\boldsymbol{n},\boldsymbol{a})P(\boldsymbol{n},\boldsymbol{a}|t^*)\}\, P(\boldsymbol{n}|r,\boldsymbol{a})P(\boldsymbol{a}|t^*) \\
&= \textstyle\sum_{\boldsymbol{a},\boldsymbol{b},\boldsymbol{n}} \begin{pmatrix} E[D|\boldsymbol{b},c^*,z,r,\boldsymbol{n},\boldsymbol{a}]P(\boldsymbol{b}|c^*,z,r,\boldsymbol{n},\boldsymbol{a})P(\boldsymbol{n}|c^*,z,r,\boldsymbol{a})P(\boldsymbol{a}|c^*,z,r) \\ \times \frac{\sum_{\boldsymbol{n},\boldsymbol{a}}\{P(\boldsymbol{b}|c^*,z,t^*,\boldsymbol{n},\boldsymbol{a})P(\boldsymbol{n},\boldsymbol{a}|t^*)\}}{P(\boldsymbol{b}|c^*,z,r,\boldsymbol{n},\boldsymbol{a})} \frac{P(\boldsymbol{n}|r,\boldsymbol{a})}{P(\boldsymbol{n}|c^*,z,r,\boldsymbol{a})} \frac{P(\boldsymbol{a}|t^*)}{(\boldsymbol{a}|c^*,r,z)} \end{pmatrix} \\
&= \textstyle\sum_{\boldsymbol{a},\boldsymbol{b},\boldsymbol{n}} \begin{pmatrix} E[D|\boldsymbol{b},c^*,z,r,\boldsymbol{n},\boldsymbol{a}]P(\boldsymbol{b}|c^*,z,r,\boldsymbol{n},\boldsymbol{a})P(\boldsymbol{n}|c^*,z,r,\boldsymbol{a})P(\boldsymbol{a}|c^*,z,r) \\ \times \frac{\sum_{\boldsymbol{n},\boldsymbol{a}}\{E[I(\boldsymbol{b})|c^*,z,t^*,\boldsymbol{n},\boldsymbol{a}]P(\boldsymbol{n},\boldsymbol{a}|t^*)\}}{E[I(\boldsymbol{b})|c^*,z,r,\boldsymbol{n},\boldsymbol{a}]} \frac{P(c^*|z,r)}{P(c^*|z,r,\boldsymbol{n},\boldsymbol{a})} \frac{P(z|r)}{P(z|r,\boldsymbol{n},\boldsymbol{a})} \frac{P(t^*|\boldsymbol{a})}{P(r|\boldsymbol{a})} \frac{P(r)}{P(t^*)} \end{pmatrix} \\
&= \textstyle\sum_{\boldsymbol{a},\boldsymbol{b},\boldsymbol{n}} \begin{pmatrix} E[D|\boldsymbol{b},c^*,z,r,\boldsymbol{n},\boldsymbol{a}]P(\boldsymbol{b}|c^*,z,r,\boldsymbol{n},\boldsymbol{a})P(\boldsymbol{n}|c^*,z,r,\boldsymbol{a})P(\boldsymbol{a}|c^*,z,r) \\ \times \frac{E\llbracket E[I(\boldsymbol{b})|c^*,z,t^*,\boldsymbol{n},\boldsymbol{a}]|t^*\rrbracket}{E[I(\boldsymbol{b})|c^*,z,r,\boldsymbol{n},\boldsymbol{a}]} \frac{P(c^*|z,r)}{P(c^*|z,r,\boldsymbol{n},\boldsymbol{a})} \frac{P(z|r)}{P(z|r,\boldsymbol{n},\boldsymbol{a})} \frac{P(t^*|\boldsymbol{a})}{P(r|\boldsymbol{a})} \frac{P(r)}{P(t^*)} \end{pmatrix} \\
&= E[W_{r,z}^{SATE-D} \times W_c \times W_b \times D|c^*,z,r]
\end{aligned}
$$

where $W_{r,z}^{SATE-D} = \frac{P(z|r)}{P(z|r,\boldsymbol{n},\boldsymbol{a})} \frac{P(t^*|a)}{P(r|a)} \frac{P(r)}{P(t^*)}$, $W_c = \frac{P(c^*|z,r)}{P(c^*|z,r,\boldsymbol{n},\boldsymbol{a})}$,
and $W_b = \frac{E\llbracket E[I(\boldsymbol{b})|c^*,z,t^*,\boldsymbol{n},\boldsymbol{a}]|t^*\rrbracket}{E[I(\boldsymbol{b})|c^*,z,r,\boldsymbol{n},\boldsymbol{a}]}$ wherein $E\llbracket\cdot\rrbracket$ is over $P(\boldsymbol{n},\boldsymbol{a}|t^*)$ and $I(\boldsymbol{b})$ is the indicator function $I(\boldsymbol{B}=\boldsymbol{b})$ which is equal to one if true and zero otherwise. (24)

The second equality bayes rule, simplification, and by noting that $P(X=x)=E[I(X=x)]$. The third equality holds by definition of an iterated conditional expectation.

Proof of G-computation Estimator for (22)

Lemma 1.

$$
\begin{aligned}
&P(G=\boldsymbol{b}|t^*) = P\left(B^{(z,c^*)}=\boldsymbol{b}\middle|t^*\right) \\
&= \textstyle\sum_{\boldsymbol{n}}\{P(\boldsymbol{b}|c^*,z,t^*,\boldsymbol{n},\boldsymbol{a})P(\boldsymbol{n},\boldsymbol{a}|t^*)\} \\
&= \textstyle\sum_{\boldsymbol{n}}\{E(I(\boldsymbol{b})|c^*,z,t^*,\boldsymbol{n},\boldsymbol{a})P(\boldsymbol{n},\boldsymbol{a}|t^*)\} \\
&= \textstyle\sum_{\boldsymbol{n}}\left\{E(I(\boldsymbol{b})|c^*,z,t^*,\boldsymbol{n},\boldsymbol{a})P(\boldsymbol{n},\boldsymbol{a}|c^*,z,t^*)\frac{P(\boldsymbol{n},\boldsymbol{a}|t^*)}{P(\boldsymbol{n},\boldsymbol{a}|c^*,z,t^*)}\right\} \\
&= \textstyle\sum_{\boldsymbol{n}}\left\{E(I(\boldsymbol{b})|c^*,z,t^*,\boldsymbol{n},\boldsymbol{a})P(\boldsymbol{n},\boldsymbol{a}|c^*,z,t^*)\frac{P(c^*|z,t^*)}{P(c^*|z,t^*,\boldsymbol{n},\boldsymbol{a})}\frac{P(z|t^*)}{P(z|t^*,\boldsymbol{n},\boldsymbol{a})}\right\}
\end{aligned}
$$

$= E[\omega \times I(\boldsymbol{b})|c^*,z,t^*]$ where $\omega = \frac{P(c^*|z,t^*)}{P(c^*|z,t^*,\boldsymbol{n},\boldsymbol{a})}\frac{P(z|t^*)}{P(z|t^*,\boldsymbol{n},\boldsymbol{a})}$, which we denote $\mathbb{P}(\boldsymbol{b}|t^*)$. Now, by definition of G as a random assignment of $\boldsymbol{B}^{(z,c^*)}$, $P(G=\boldsymbol{b}|t^*) = P(G=\boldsymbol{b}|t^*,\boldsymbol{n},\boldsymbol{a}) \Rightarrow$ $P\left(B^{(z,c^*)}=\boldsymbol{b}\middle|t^*\right) = P\left(\boldsymbol{B}^{(z,c^*)}=\boldsymbol{b}\middle|t^*,\boldsymbol{n},\boldsymbol{a}\right) \Rightarrow \mathbb{P}(\boldsymbol{b}|t^*) = \mathbb{P}(\boldsymbol{b}|t^*,\boldsymbol{n},\boldsymbol{a})$.

This concludes Lemma 1. Now we have that:

$$
\begin{aligned}
&\textstyle\sum_{\boldsymbol{a},\boldsymbol{b},\boldsymbol{n}} E[D|\boldsymbol{b},c^*,z,r,\boldsymbol{n},\boldsymbol{a}] \sum_{\boldsymbol{n},\boldsymbol{a}}\{P(\boldsymbol{b}|c^*,z,t^*,\boldsymbol{n},\boldsymbol{a})P(\boldsymbol{n},\boldsymbol{a}|t^*)\}\, P(\boldsymbol{n}|r,\boldsymbol{a})P(\boldsymbol{a}|t^*) \\
&= \textstyle\sum_{\boldsymbol{a},\boldsymbol{b},\boldsymbol{n}} E[D|\boldsymbol{b},c^*,z,r,\boldsymbol{n},\boldsymbol{a}]\mathbb{P}(\boldsymbol{b}|t^*,\boldsymbol{n},\boldsymbol{a})\boldsymbol{P}(\boldsymbol{n}|r,\boldsymbol{a})P(\boldsymbol{a}|t^*) \\
&= \textstyle\sum_{\boldsymbol{a},\boldsymbol{n}} E\{E[D|\boldsymbol{b},c^*,z,r,\boldsymbol{n},\boldsymbol{a}]|z,t^*,\boldsymbol{n},\boldsymbol{a}\}P(\boldsymbol{n}|r,\boldsymbol{a})P(\boldsymbol{a}|t^*) \\
&= \textstyle\sum_{\boldsymbol{a}} E(E\{E[D|\boldsymbol{b},c^*,z,r,\boldsymbol{n},\boldsymbol{a}]|z,t^*,\boldsymbol{n},\boldsymbol{a}\}|r,\boldsymbol{a})P(\boldsymbol{a}|t^*) \\
&= E\llbracket E(E\{E[D|\boldsymbol{b},c^*,z,r,\boldsymbol{n},\boldsymbol{a}]|z,t^*,\boldsymbol{n},\boldsymbol{a}\}|r,\boldsymbol{a})|t^*\rrbracket
\end{aligned} \tag{25}
$$

where $E\{\cdot\}$ is over $P\left(B^{(z,c^*)}=\boldsymbol{b}\middle|t^*\right)$ which is identified by $\mathbb{P}(\boldsymbol{b}|t^*,\boldsymbol{n},\boldsymbol{a})$ as defined above, $E(\cdot)$ is over $P(\boldsymbol{n}|r,\boldsymbol{a})$, and $E\llbracket\cdot\rrbracket$ is over $P(\boldsymbol{a}|t^*)$.

The first equality holds by Lemma 1, and the remaining by definition of an iterated expectation.

Sample Interventional Direct Effect of Treatment on Disparity Among the Treated (SITT-D)

Estimand

$$
\begin{aligned}
&\ddot{\mu}^{z}(r) \\
&= \textstyle\sum_{\boldsymbol{a}} E\left[D^{(z,c^*,\boldsymbol{G}=\boldsymbol{b})} \middle| z^*, r, \boldsymbol{a}\right] P(\boldsymbol{a}|z^*, t^*) \\
&= \textstyle\sum_{\boldsymbol{a},\boldsymbol{b}} E\left[D^{(z,c^*,\boldsymbol{G}=\boldsymbol{b})} \middle| \boldsymbol{G}=\boldsymbol{b}, z^*, r, \boldsymbol{a}\right] P(\boldsymbol{G}=\boldsymbol{b}|z^*, r, \boldsymbol{a}) P(\boldsymbol{a}|z^*, t^*) \qquad (26)
\end{aligned}
$$

Identifying Formula for (26)

$$
\begin{aligned}
&\textstyle\sum_{\boldsymbol{a},\boldsymbol{b}} E\left[D^{(z,c^*,\boldsymbol{G}=\boldsymbol{b})} \middle| \boldsymbol{G}=\boldsymbol{b}, z^*, r, \boldsymbol{a}\right] P(\boldsymbol{G}=\boldsymbol{b}|z^*, r, \boldsymbol{a}) P(\boldsymbol{a}|z^*, t^*) \\
&= \textstyle\sum_{\boldsymbol{a},\boldsymbol{b}} E\left[D^{(z,c^*,\boldsymbol{b})} \middle| z^*, r, \boldsymbol{a}\right] P(\boldsymbol{G}=\boldsymbol{b}|z^*, r, \boldsymbol{a}) P(\boldsymbol{a}|z^*, t^*) \\
&= \textstyle\sum_{\boldsymbol{a},\boldsymbol{b},\boldsymbol{n}} E\left[D^{(z,c^*,\boldsymbol{b})} \middle| z^*, r, \boldsymbol{n}, \boldsymbol{a}\right] P(\boldsymbol{G}=\boldsymbol{b}|z^*, r, \boldsymbol{n}, \boldsymbol{a}) P(\boldsymbol{n}|z^*, r, \boldsymbol{a}) P(\boldsymbol{a}|z^*, t^*) \\
&= \textstyle\sum_{\boldsymbol{a},\boldsymbol{b},\boldsymbol{n}} E\left[D^{(z,c^*,\boldsymbol{b})} \middle| z^*, r, \boldsymbol{n}, \boldsymbol{a}\right] P(\boldsymbol{G}=\boldsymbol{b}|z^*, r, \boldsymbol{a}) P(\boldsymbol{n}|z^*, r, \boldsymbol{a}) P(\boldsymbol{a}|z^*, t^*) \\
&= \textstyle\sum_{\boldsymbol{a},\boldsymbol{b},\boldsymbol{n}} E\left[D^{(z,c^*,\boldsymbol{b})} \middle| c^*, z, r, \boldsymbol{n}, \boldsymbol{a}\right] P(\boldsymbol{G}=\boldsymbol{b}|z^*, r, \boldsymbol{a}) P(\boldsymbol{n}|z^*, r, \boldsymbol{a}) P(\boldsymbol{a}|z^*, t^*) \\
&= \textstyle\sum_{\boldsymbol{a},\boldsymbol{b},\boldsymbol{n}} E\left[D^{(z,c^*,\boldsymbol{b})} \middle| \boldsymbol{b}, c^*, z, r, \boldsymbol{n}, \boldsymbol{a}\right] P(\boldsymbol{G}=\boldsymbol{b}|z^*, r, \boldsymbol{a}) P(\boldsymbol{n}|z^*, r, \boldsymbol{a}) P(\boldsymbol{a}|z^*, t^*) \\
&= \textstyle\sum_{\boldsymbol{a},\boldsymbol{b},\boldsymbol{n}} E[D|\boldsymbol{b}, c^*, z, r, \boldsymbol{n}, \boldsymbol{a}] P(\boldsymbol{G}=\boldsymbol{b}|z^*, r, \boldsymbol{a}) P(\boldsymbol{n}|z^*, r, \boldsymbol{a}) P(\boldsymbol{a}|z^*, t^*) \\
&= \textstyle\sum_{\boldsymbol{a},\boldsymbol{b},\boldsymbol{n}} E[D|\boldsymbol{b}, c^*, z, r, \boldsymbol{n}, \boldsymbol{a}] P\left(\boldsymbol{B}^{(z,c^*)}=\boldsymbol{b} \middle| z^*, t^*\right) P(\boldsymbol{n}|z^*, r, \boldsymbol{a}) P(\boldsymbol{a}|z^*, t^*) \\
&= \textstyle\sum_{\boldsymbol{a},\boldsymbol{b},\boldsymbol{n}} \begin{pmatrix} E[D|\boldsymbol{b}, c^*, z, r, \boldsymbol{n}, \boldsymbol{a}] \\ \times \sum_{\boldsymbol{n},\boldsymbol{a}} \left\{P\left(\boldsymbol{B}^{(z,c^*)}=\boldsymbol{b} \middle| z^*, t^*, \boldsymbol{n}, \boldsymbol{a}\right) P(\boldsymbol{n}, \boldsymbol{a}|z^*, t^*)\right\} \\ \times P(\boldsymbol{n}|z^*, r, \boldsymbol{a}) P(\boldsymbol{a}|z^*, t^*) \end{pmatrix} \\
&= \textstyle\sum_{\boldsymbol{a},\boldsymbol{b},\boldsymbol{n}} \begin{pmatrix} E[D|\boldsymbol{b}, c^*, z, r, \boldsymbol{n}, \boldsymbol{a}] \\ \times \sum_{\boldsymbol{n},\boldsymbol{a}} \left\{P\left(\boldsymbol{B}^{(z,c^*)}=\boldsymbol{b} \middle| c^*, z, t^*, \boldsymbol{n}, \boldsymbol{a}\right) P(\boldsymbol{n}, \boldsymbol{a}|z^*, t^*)\right\} \\ \times P(\boldsymbol{n}|z^*, r, \boldsymbol{a}) P(\boldsymbol{a}|z^*, t^*) \end{pmatrix} \\
&= \\
&\textstyle\sum_{\boldsymbol{a},\boldsymbol{b},\boldsymbol{n}} E[D|\boldsymbol{b}, c^*, z, r, \boldsymbol{n}, \boldsymbol{a}] \sum_{\boldsymbol{n},\boldsymbol{a}} \{P(\boldsymbol{b}|c^*, z, t^*, \boldsymbol{n}, \boldsymbol{a}) P(\boldsymbol{n}, \boldsymbol{a}|z^*, t^*)\} P(\boldsymbol{n}|z^*, r, \boldsymbol{a}) P(\boldsymbol{a}|z^*, t^*) \qquad (27)
\end{aligned}
$$

The expression on the LHS of the first line is the estimand and holds by the overlap assumptions A1*, C1* (D1* under study attrition), and the total law of probability. (Overlap C1* (D1* under study attrition) itself requires assumptions C2*, A3*, and C4* [D2*, B2*, and D4* under study attrition]). The first equality holds by definition of the intervention $\boldsymbol{G}$ as random given $\boldsymbol{R}=\boldsymbol{r}$ and $\boldsymbol{A}=\boldsymbol{a}$, the second by the total law of probability, the third by definition of the intervention $\boldsymbol{G}$ as a random assignment, the fourth by conditional exchangeability A2* (and B1* under study attrition), and positivity A3* (and B2* under study attrition), and the fifth by conditional exchangeability C2* (D2* under study attrition) and positivity C3* (D3*) under study attrition, the sixth by consistency C4* (D4* under study attrition), the seventh by definition of the intervention $\boldsymbol{G}$ as a random draw from the conditional counterfactual distribution of $\boldsymbol{B}$ under assignment of Z to value z among the standard population $T=t^*$ and the treated arm $Z=z^*$, the eighth by the total law of probability, the ninth by conditional exchangeability C2* (D2* under study attrition) and positivity A3* (and B2* under study attrition), and the tenth by consistency C4* (D4* under study attrition). For the SITT-D the standard population $T=t^*$ is chosen among the treated arm $Z=z^*$.

Proof of Weighting Estimator for (26)

$$\begin{aligned}
&\sum_{\boldsymbol{a},\boldsymbol{b},\boldsymbol{n}} E[D_k|\boldsymbol{b},c^*,z,r,\boldsymbol{n},\boldsymbol{a}]\sum_{\boldsymbol{n}}\{P(\boldsymbol{b}|c^*,z,t^*,\boldsymbol{n},\boldsymbol{a})P(\boldsymbol{n},\boldsymbol{a}|z^*,t^*)\}\,P(\boldsymbol{n}|z^*r,\boldsymbol{a})P(\boldsymbol{a}|z^*,t^*)\\
&=\sum_{\boldsymbol{a},\boldsymbol{b},\boldsymbol{n}}\left(\begin{array}{c}E[D_k|\boldsymbol{b_k},c^*,z,r,\boldsymbol{n},\boldsymbol{a}]P(\boldsymbol{b}|c^*,z,r,\boldsymbol{n},\boldsymbol{a})P(\boldsymbol{n}|c^*,z,r,\boldsymbol{a})P(\boldsymbol{a}|c^*,z,r)\\ \times\frac{\sum_{n,a}\{P(\boldsymbol{b}|c^*,z,t^*,\boldsymbol{n},\boldsymbol{a})P(\boldsymbol{n},\boldsymbol{a}|z^*,t^*)\}}{P(\boldsymbol{b}|c^*,z,r,\boldsymbol{n},\boldsymbol{a})}\frac{P(\boldsymbol{n}|z^*,r,\boldsymbol{a})}{P(\boldsymbol{n}|c^*,z,r,\boldsymbol{a})}\frac{P(\boldsymbol{a}|z^*,t^*)}{(\boldsymbol{a}|c^*,z,r)}\end{array}\right)\\
&=\sum_{\boldsymbol{a},\boldsymbol{b},\boldsymbol{n}}\left(\begin{array}{c}E[D|\boldsymbol{b},c^*,z,r,\boldsymbol{n},\boldsymbol{a}]P(\boldsymbol{b}|c^*,z,r,\boldsymbol{n},\boldsymbol{a})P(\boldsymbol{n}|c^*,z,r,\boldsymbol{a})P(\boldsymbol{a}|c^*,z,r)\\ \times\frac{E[\![E[I(\boldsymbol{b}|c^*,z,t^*,\boldsymbol{n},\boldsymbol{a}]|z^*,t^*]\!]}{E[I(\boldsymbol{b})|c^*,z,r,\boldsymbol{n},\boldsymbol{a}]}\frac{P(c^*|z,r)}{P(c^*|z,r,\boldsymbol{n},\boldsymbol{a})}\frac{P(z^*|r,\boldsymbol{n},\boldsymbol{a})}{P(z|r,\boldsymbol{n},\boldsymbol{a})}\frac{P(t^*,z^*|\boldsymbol{a})}{P(r,z^*|\boldsymbol{a})}\frac{P(r,z)}{P(t^*,z^*)}\end{array}\right)\\
&=E[W_{r,z}^{SATT-D}\times W_c\times W_b\times D|c^*,z,r]
\end{aligned}$$

where $W_{r,z}^{SATT-D}=\frac{P(z^*|r,\boldsymbol{n},\boldsymbol{a})}{P(z|r,\boldsymbol{n},\boldsymbol{a})}\frac{P(t^*,z^*|\boldsymbol{a})}{P(r,z^*|\boldsymbol{a})}\frac{P(r,z)}{P(t^*,z^*)}$, $W_c=\frac{P(c^*|z,r)}{P(c^*|z,r,\boldsymbol{n},\boldsymbol{a})}$,

and $W_b=\frac{E[\![E[I(\boldsymbol{b})|c^*,z,t^*,\boldsymbol{n},\boldsymbol{a}]|z^*,t^*]\!]}{E[I(\boldsymbol{b})|c^*,z,r,\boldsymbol{n},\boldsymbol{a}]}$ wherein $E[\![\cdot]\!]$ is over $P(\boldsymbol{n},\boldsymbol{a}|z^*,t^*)$ and $I(\boldsymbol{b})$ is the indicator function $I(\boldsymbol{B}=\boldsymbol{b})$ which is equal to one if true and zero otherwise. (28)

The second equality holds by bayes rule, some simplification, and by also noting that $P(X=x)=E[I(X=x)]$ and by the definition of an iterated conditional expectation.

Proof of G-computation Estimator for (26)

Lemma 2.

$$\begin{aligned}
&P(G=\boldsymbol{b}|z^*,t^*)=P\left(B^{(z,c^*)}=\boldsymbol{b}\middle|z^*,t^*\right)\\
&=\sum_{\boldsymbol{n}}\{P(\boldsymbol{b}|c^*,z,t^*,\boldsymbol{n},\boldsymbol{a})P(\boldsymbol{n},\boldsymbol{a}|z^*,t^*)\}\\
&=\sum_{\boldsymbol{n}}\left\{E(I(\boldsymbol{b})|c^*,z,t^*,\boldsymbol{n},\boldsymbol{a})P(\boldsymbol{n},\boldsymbol{a}|c^*,z,t^*)\frac{P(\boldsymbol{n},\boldsymbol{a}|z^*,t^*)}{P(\boldsymbol{n},\boldsymbol{a}|c^*,z,t^*)}\right\}\\
&=\sum_{\boldsymbol{n}}\left\{E(I(\boldsymbol{b})|c^*,z,t^*,\boldsymbol{n},\boldsymbol{a})P(\boldsymbol{n},\boldsymbol{a}|c^*,z,t^*)\frac{P(c^*|z,t^*,\boldsymbol{a})}{P(c^*|z,t^*,\boldsymbol{n},\boldsymbol{a})}\frac{P(z^*|t^*,\boldsymbol{n},\boldsymbol{a})}{P(z|t^*,\boldsymbol{n},\boldsymbol{a})}\frac{P(t^*,z)}{P(t^*,z^*)}\right\}\\
&=E[\omega\times I(\boldsymbol{b})|c^*,z,t^*]\text{ where }\omega=\frac{P(c^*|z,t^*)}{P(c^*|z,t^*,\boldsymbol{n},\boldsymbol{a})}\frac{P(z^*|t^*,\boldsymbol{n},\boldsymbol{a})}{P(z|t^*,\boldsymbol{n},\boldsymbol{a})}\frac{P(t^*,z)}{P(t^*,z^*)},
\end{aligned}$$

which we denote $\mathbb{P}(\boldsymbol{b}|z^*,t^*)$. By definition of G as a random assignment of $\boldsymbol{B}^{(z,c^*)}$, we have $P(G=\boldsymbol{b}|z^*,t^*)=P(G=\boldsymbol{b}|z^*,t^*,\boldsymbol{n},\boldsymbol{a})\Rightarrow P\left(B^{(z,c^*)}=\boldsymbol{b}\middle|z^*,t^*\right)=P\left(B^{(z,c^*)}=\boldsymbol{b}\middle|z^*,t^*,\boldsymbol{n},\boldsymbol{a}\right)$

$\Rightarrow\mathbb{P}(\boldsymbol{b}|z^*,t^*)=\mathbb{P}(\boldsymbol{b}|z^*,t^*,\boldsymbol{n},\boldsymbol{a})$.

This concludes Lemma 2.

Now we have that:

$$\begin{aligned}
&\sum_{\boldsymbol{a},\boldsymbol{b},\boldsymbol{n}}E[D|\boldsymbol{b},c^*,z,r,\boldsymbol{n},\boldsymbol{a}]\sum_{\boldsymbol{n},\boldsymbol{a}}\{P(\boldsymbol{b}|c^*,z,t^*,\boldsymbol{n},\boldsymbol{a})P(\boldsymbol{n},\boldsymbol{a}|z^*,t^*)\}\,P(\boldsymbol{n}|z^*,r,\boldsymbol{a})P(\boldsymbol{a}|z^*,t^*)\\
&=\sum_{\boldsymbol{a},\boldsymbol{b},\boldsymbol{n}}E[D|\boldsymbol{b_k},c^*,z,r,\boldsymbol{n},\boldsymbol{a}]\mathbb{P}(\boldsymbol{b}|z^*,t^*,\boldsymbol{n},\boldsymbol{a})P(\boldsymbol{n}|z^*,r,\boldsymbol{a})P(\boldsymbol{a}|z^*,t^*)\\
&=\sum_{\boldsymbol{a},\boldsymbol{n}}E\{E[D|\boldsymbol{b_k},c^*,z,r,\boldsymbol{n},\boldsymbol{a}]|z,t^*,\boldsymbol{n},\boldsymbol{a}\}P(\boldsymbol{n}|z^*,r,\boldsymbol{a})P(\boldsymbol{a}|z^*,t^*)\\
&=\sum_{\boldsymbol{a}}E(E\{E[D|\boldsymbol{b_k},c^*,z,r,\boldsymbol{n},\boldsymbol{a}]|z,t^*,\boldsymbol{n},\boldsymbol{a}\}|z^*,r,\boldsymbol{a})P(\boldsymbol{a}|z^*,t^*)\\
&=E[\![E(E\{E[D|\boldsymbol{b_k},c^*,z,r,\boldsymbol{n},\boldsymbol{a}]|z,t^*,\boldsymbol{n},\boldsymbol{a}\}|z^*,r,\boldsymbol{a})|z^*,t^*]\!]
\end{aligned}\qquad(29)$$

where $E\{\cdot\}$ is over $P\left(\boldsymbol{B}^{(z,c^*)}=\boldsymbol{b}\middle|z^*,t^*\right)$ which is identified by $\mathbb{P}(\boldsymbol{b}|z^*,t^*\boldsymbol{n},\boldsymbol{a})$ as defined above, $E(\cdot)$ is over $P(\boldsymbol{n}|z^*,r,\boldsymbol{a})$, and $E[\![\cdot]\!]$ is over $P(\boldsymbol{a}|z^*,t^*)$.

The first equality holds by Lemma 1, and the remaining by definition of an iterated expectation.

References

Asada, Y. (2010). On the choice of absolute or relative inequality measures. *Milbank Q.,* 88, 616-622; discussion 623-617.

Bailey, Z.D., Krieger, N., Agénor, M., Graves, J., Linos, N., & Bassett, M.T. (2017). Structural racism and health inequities in the USA: evidence and interventions. *Lancet,* 389, 1453-1463.

Colantuoni, E., & Rosenblum, M. (2015). Leveraging prognostic baseline variables to gain precision in randomized trials. *Stat. Med.,* 34, 2602-2617.

Davison, A.C., Hinkley, D.V., & Schechtman, E. (1986). Efficient Bootstrap Simulation. *Biometrika,* 73, 555.

Deen, M., & de Rooij, M. (2020). ClusterBootstrap: An R package for the analysis of hierarchical data using generalized linear models with the cluster bootstrap. *Behav. Res. Methods,* 52, 572-590.

Didelez, V., Dawid, P., & Geneletti, S. (2006). Direct and Indirect Effects of Sequential Treatments. UAI'06: Proceedings of the Twenty-Second Conference on Uncertainty in Artificial Intelligence pp. 138-146).

Efron, B., & Tibshirani, R. (1986). Bootstrap Methods for Standard Errors, Confidence Intervals, and Other Measures of Statistical Accuracy. *Stat. Sci.,* 1, 54-75.

Field, C.A., & Welsh, A.H. (2007). Bootstrapping clustered data. *J. R. Stat. Soc. Series B Stat. Methodol.,* 69, 369-390.

Geneletti, S. (2007). Identifying direct and indirect effects in a non-counterfactual framework. *J. R. Stat. Soc. Series B Stat. Methodol.,* 69, 199-215.

Gleason, J.R. (1988). Algorithms for Balanced Bootstrap Simulations. *Am. Stat.,* 42, 263-266.

Greenland, S., & Mansournia, M.A. (2015). Limitations of individual causal models, causal graphs, and ignorability assumptions, as illustrated by random confounding and design unfaithfulness. *Eur. J. Epidemiol.,* 30, 1101-1110.

Hernán, M.A., & Robins, J.M. (2006). Estimating causal effects from epidemiological data. *J. Epidemiol. Community Health,* 60, 578-586.

Hernán, M.A., & Robins, J.M. (2020). *Causal Inference: What If*. Boca Raton: Chapman & Hall/CRC.

Huang, F.L. (2018). Using Cluster Bootstrapping to Analyze Nested Data With a Few Clusters. *Educ. Psychol. Meas.,* 78, 297-318.

Kjellsson, G., Gerdtham, U.-G., & Petrie, D. (2015). Lies, Damned Lies, and Health Inequality Measurements: Understanding the Value Judgments. *Epidemiology,* 26, 673-680.

Landau, S., & Stahl, D. (2013). Sample size and power calculations for medical studies by simulation when closed form expressions are not available. *Stat. Methods Med. Res.,* 22, 324-345.

Muñoz, I.D., & van der Laan, M. (2012). Population intervention causal effects based on stochastic interventions. *Biometrics,* 68, 541-549.

Neyman, J. (1923). On the application of probability theory to agricultural experiments. Essay on principles. *Ann. Agricultural Sciences*, 1-51.

Ren, S., Lai, H., Tong, W., Aminzadeh, M., Hou, X., & Lai, S. (2010). Nonparametric bootstrapping for hierarchical data. *J. Appl. Stat.,* 37, 1487-1498.

Robins, J.M. (1986). A new approach to causal inference in mortality studies with a sustained exposure period—application to control of the healthy worker survivor effect. *Mathematical Modelling,* 7, 1393-1512.

Rubin, D.B. (1974). Estimating causal effects of treatments in randomized and nonrandomized studies. *J. Educ. Psychol.,* 66, 688-701.

Sobel, M.E. (2006). What do randomized studies of housing mobility demonstrate?: Causal inference in the face of interference. *Journal of the American Statistical Association,* 94, 1398-1407.

VanderWeele, T.J. (2009). Concerning the consistency assumption in causal inference. *Epidemiology,* 20, 880-883.

VanderWeele, T.J., & Robins, J.M. (2007). Four types of effect modification: a classification based on directed acyclic graphs. *Epidemiology,* 18, 561-568.

Zivich, P.N., Cole, S.R., & Westreich, D. (2022). Positivity: Identifiability and Estimability. *arXiv [stat.ME].*

Example Analysis Code

```
#### DESCRIPTION OF mydata DATAFRAME ####
#pid – person id
#sid – site id
#A - allowable
#N - non-allowable
#R – social group (1=marginalized,0=privileged)
#TT- membership in standard population (1=yes,0=no)
#Z – intervention arm (1=treatment,0=control)
#C – loss to follow-up (1=yes,0=no)
#Y – health outcome, e.g., hypertension control (also a post-intervention allowable for D)
#D – decision outcome, e.g., treatment intensification

##### FUNCTION TO CARRY OUT SATE WEIGHTING #####
wgt_sate <- function(df,outcome,in_weight=NULL,check_weights=FALSE) {
  if (!is.null(in_weight)) {
    df$W_extra <- in_weight
    } else {
      df$W_extra<-1
      }
  #Step 0: pull outcome
  df[,"Y"] <- unlist(df[,outcome])
  #Step 1: fit models
  fit_r    <- glm(R~1,binomial("logit"),df)
  fit_rx   <- glm(R~A,binomial("logit"),df)
  fit_t    <- glm(TT~1,binomial("logit"),df)
  fit_tx   <- glm(TT~A,binomial("logit"),df)
  fit_zr   <- glm(Z~R,binomial("logit"),df)
  fit_zrx  <- glm(Z~R+N+A,binomial("logit"),df)
  fit_czr  <- glm(C~Z+R+Z*R,binomial("logit"),df) #comment out if no loss to follow-up
  fit_czrx <- glm(C~Z+R+Z*R+N+A,binomial("logit"),df) #comment out if no loss to follow-up

  #Step 2: obtain predicted values
  df[,"p_r"]    <- predict(fit_r,df,"response")
  df[,"p_rx"]   <- predict(fit_rx,df,"response")
  df[,"p_t"]    <- predict(fit_t,df,"response")
  df[,"p_tx"]   <- predict(fit_tx,df,"response")
  df[,"p_zr"]   <- predict(fit_zr,df,"response")
  df[,"p_zrx"]  <- predict(fit_zrx,df,"response")
  df[,"p_czr"]  <- predict(fit_czr,df,"response")  # comment out if no loss to follow-up
  df[,"p_czrx"] <- predict(fit_czrx,df,"response")   #comment out if no loss to follow-up

  #Step 3: calculate predicted values
  df[,"p_r"]    <- df$R*df$p_r+(1-df$R)*(1-df$p_r)
  df[,"p_rx"]   <- df$R*df$p_rx+(1-df$R)*(1-df$p_rx)
  df[,"p_zr"]   <- df$Z*df$p_zr+(1-df$Z)*(1-df$p_zr)
  df[,"p_zrx"]  <- df$Z*df$p_zrx+(1-df$Z)*(1-df$p_zrx)
  df[,"p_czr"]  <- df$C*df$p_czr+(1-df$C)*(1-df$p_czr) #comment out if no loss to follow-up
  df[,"p_czrx"] <- df$C*df$p_czrx+(1-df$C)*(1-df$p_czrx) #comment out if no loss to follow-up

  #Step 4: create weights
  df[,"W_zr"] <- (df$p_r*df$p_tx)/(df$p_t*df$p_rx) * df$p_zr/df$p_zrx * df$W_extra *
df$p_czr/df$p_czrx #comment out last part if no loss to follow-up
  if (isTRUE(check_weights)) {
    weights<-df$W_zr
    weights_distribution<-
c(mean(weights,na.rm=TRUE),min(weights,na.rm=TRUE),max(weights,na.rm=TRUE))
    print(weights_distribution)
    }
  #step 5: estimate effect on disparity by contrasting weighted averages
  dfZ1R1 <- df[which(df$Z==1 & df$R==1),]#& df$C==0),]
  dfZ0R1 <- df[which(df$Z==0 & df$R==1),]#& df$C==0),]
  dfZ1R0 <- df[which(df$Z==1 & df$R==0),]#& df$C==0),]
  dfZ0R0 <- df[which(df$Z==0 & df$R==0),]#& df$C==0),]
  Y11 <- weighted.mean(dfZ1R1$Y,dfZ1R1$W_zr)
  Y01 <- weighted.mean(dfZ0R1$Y,dfZ0R1$W_zr)
  Y10 <- weighted.mean(dfZ1R0$Y,dfZ1R0$W_zr)
  Y00 <- weighted.mean(dfZ0R0$Y,dfZ0R0$W_zr)
```

```
  RD <- (Y11-Y01)-(Y10-Y00)
  RR <- (Y11/Y01)/(Y10/Y00)
  output<-c(Y11,Y01,Y10,Y00,RD,RR)
  names(output) <- c("Z1R1","Z0R1","Z1R0","Z0R0","RD","RR")
  return(output)
  }

##### FUNCTION TO CARRY OUT SITE WEIGHTING #####
wgt_site <- function(df,outcome) {
  ZR_mat<-matrix(c(1,1,0,1,1,0,0,0),4,2,TRUE)
  list_pred_b_r<-list()
  list_pred_b_t<-list()
  for (iter in 1:4) {
    zval<-ZR_mat[iter,1]
    rval<-ZR_mat[iter,2]
    fit_b_r <- glm(Y~N+A,binomial("logit"),df[which(df$Z==zval & df$R==rval),])
    list_pred_b_r[[iter]] <- predict(fit_b_r,df,"response")
    }
  Z_vec<-c(1,0)
  for (jiter in 1:2) {
    zval<-Z_vec[jiter]
    fit_b_t  <- glm(Y~N+A,binomial("logit"),df[which(df$Z==zval & df$TT==1),])
    pred_b_t <- predict(fit_b_t,df[which(df$TT==1),],"response")
    list_pred_b_t[[jiter]]<-I(df$Y==1)*mean(pred_b_t)+I(df$Y==0)*(mean(1-pred_b_t))
    }
  df[,"pbx_zr11"] <- list_pred_b_r[[1]]*(I(df$Z==1 & df$R==1))
  df[,"pbx_zr01"] <- list_pred_b_r[[2]]*(I(df$Z==0 & df$R==1))
  df[,"pbx_zr10"] <- list_pred_b_r[[3]]*(I(df$Z==1 & df$R==0))
  df[,"pbx_zr00"] <- list_pred_b_r[[4]]*(I(df$Z==0 & df$R==0))
  df[,"pbx_zt11"] <- list_pred_b_t[[1]]*I(df$Z==1)
  df[,"pbx_zt01"] <- list_pred_b_t[[2]]*I(df$Z==0)
  df[,"pbx_r"] <- df[,"pbx_zr11"]+df[,"pbx_zr01"]+df[,"pbx_zr10"]+df[,"pbx_zr00"]
  df[,"pbx_t"] <- df[,"pbx_zt11"]+df[,"pbx_zt01"]
  df$W_b<-df$pbx_t/df$pbx_r
  output<-wgt_sate(df=df,outcome="D",in_weight=df$W_b)
  return(output)
  }

##### FUNCTION TO CARRY OUT SATE G-COMPUTATION #####
gcm_sate <- function(df,outcome) {
  #Setup iteration
  ZR_mat<-matrix(c(1,1,0,1,1,0,0,0),4,2,TRUE)
  meanTT_list<-list()
  #Step 0: pull outcome
  df[,"Y"] <- unlist(df[,outcome])
  for (iter in 1:4) {
    zval<-ZR_mat[iter,1]
    rval<-ZR_mat[iter,2]
    #Step 1: fit model for Y among Z=z & R=r
    fit_step1 <- glm(Y~N+A,quasibinomial("logit"),df[which(df$Z==zval & df$R==rval),])
    #Step 2: obtain predicted values
    df[,"Q1"] <- predict(fit_step1,df,"response")
    #Step 3: fit model for Y among R=r
    fit_step3 <- glm(Q1~A,quasibinomial("logit"),df[which(df$R==rval),])
    #Step 4: obtain predicted values
    df[,"Q2"] <- predict(fit_step3,df,"response")
    #Step 5: obtain average among standard population TT=1
    dfTT <- df[which(df$TT==1),]
    meanTT_list[[iter]]<-mean(dfTT$Q2)
    }
  Y11 <- meanTT_list[[1]]
  Y01 <- meanTT_list[[2]]
  Y10 <- meanTT_list[[3]]
  Y00 <- meanTT_list[[4]]
  RD <- (Y11-Y01)-(Y10-Y00)
  RR <- (Y11/Y01)/(Y10/Y00)
  output<-c(Y11,Y01,Y10,Y00,RD,RR)
  names(output) <- c("Z1R1","Z0R1","Z1R0","Z0R0","RD","RR")
  return(output)
  }
```

```
##### FUNCTION TO CARRY OUT SITE G-COMPUTATION #####
gcm_site <- function(df,outcome)  {
  #Setup iteration
  ZR<-matrix(c(1,1,0,1,1,0,0,0),4,2,TRUE)
  meanTT_list<-list()
  #Setup: pull outcome
  df[,"D"] <- unlist(df[,outcome])
  #Setup: create weight
  fit_zt       <- glm(Z~1,binomial("logit"),df[which(df$TT==1),])
  fit_ztx      <- glm(Z~N+A,binomial("logit"),df[which(df$TT==1),])
  df[,"p_zt"]  <- predict(fit_zt,df,"response")
  df[,"p_ztx"] <- predict(fit_ztx,df,"response")
  df[,"p_zt"]  <- df$Z*df$p_zt+(1-df$Z)*(1-df$p_zt)
  df[,"p_ztx"] <- df$Z*df$p_ztx+(1-df$Z)*(1-df$p_ztx)
  df[,"W_b"]   <- df$p_zt/df$p_ztx
  Qi_list<-list()
  Qii_list<-list()
  for (kiter in 1:4) {
    zval<-ZR[kiter,1]
    rval<-ZR[kiter,2]
    #Prelim Step i: fit model for Y among Z=z & R=r
    fit_step_i <- glm(D~Y+N+A,quasibinomial("logit"),df[which(df$Z==zval & df$R==rval),])
    #Prelim Step ii: obtain predicted values
    df[which(df$Z==zval & df$TT==1),"Qi"] <- predict(fit_step_i,df[which(df$Z==zval &
df$TT==1),],"response")
    #Prelim Step iii: fit model for Y among Z=z & T=t
    fit_step_iii <- glm(Qi~N+A,quasibinomial("logit"),df[which(df$Z==zval &
df$TT==1),],df[which(df$Z==zval & df$TT==1),"W_b"])
    #Prelim Step iv: obtain predicted values
    Qii_list[[kiter]] <- predict(fit_step_iii,df,"response")
    }
  df[,"Qii_11"]<-Qii_list[[1]]*I(df$Z==1 & df$R==1)
  df[,"Qii_01"]<-Qii_list[[2]]*I(df$Z==0 & df$R==1)
  df[,"Qii_10"]<-Qii_list[[3]]*I(df$Z==1 & df$R==0)
  df[,"Qii_00"]<-Qii_list[[4]]*I(df$Z==0 & df$R==0)
  df[,"Qii"]<-df[,"Qii_11"]+df[,"Qii_10"]+df[,"Qii_01"]+df[,"Qii_00"]
  output<-gcm_sate(df,outcome="Qii")
  return(output)
  }

##### FUNCTION TO MAKE BOOTSTRAP SAMPLE ID MATRIX #####
make_boot_id_matrix <-function (df,n_boot_samples,boot_type) {
  ctable<-table(df$H,df$R,df$Z) #clusters stratified (row) by treatment (column) and race (table)
  cnames<-rownames(ctable)
  cBWT<-cnames[apply(ctable[,,2],1,function (x) all(x>0))] #treated clusters with 0<P(R=1)<1
  cBWC<-cnames[apply(ctable[,,1],1,function (x) all(x>0))] #control clusters with 0<P(R=1)<1
  cBBT<-cnames[apply(ctable[,,2],1,function (x) x[1]==0 & x[2]>0)] #treated clusters with
P(R=1)==1
  cBBC<-cnames[apply(ctable[,,1],1,function (x) x[1]==0 & x[2]>0)] #control clusters with
P(R=1)==1
  cWWT<-cnames[apply(ctable[,,2],1,function (x) x[1]>0  & x[2]==0)] #treated clusters with
P(R=0)==1
  cWWC<-cnames[apply(ctable[,,1],1,function (x) x[1]>0  & x[2]==0)] #control clusters with
P(R=0)==1
  length(c(cBWT,cBWC,cBBT,cBBC,cWWT,cWWC))
  c_list<-list(cBWT,cBWC,cBBT,cBBC,cWWT,cWWC)
  m_list<-list()
  J<-n_boot_samples
  c_length<-lapply(c_list,length)
  c_list<-c_list[which(c_length>0)]
  if (boot_type=="balanced") {
    for (iter in 1:length(c_list)) { #For each type, create balanced matrix of cluster ids for
bootstrap samples
      cXX<-c_list[[iter]]
      vXX<-rep(cXX,J) #vector
      nXX<-runif(length(vXX),0,1) #random number
      mXX<-data.frame(vXX,nXX) #dataframe vector & random number
      sXX<-mXX[order(nXX),] #sorted dataframe by random number
      m_list[[iter]]<-matrix(sXX$vXX,length(cXX),J)
```

```
      }
  } else if (boot_type=="sampled") {
    for (iter in 1:length(c_list)) { #for each type, create matrix of cluster ids via sampling
with replacement
      cXX<-c_list[[iter]]
      mXX<-matrix(rep(cXX,J),length(cXX),J)
      sXX<-apply(mXX,2,function (x) sample(x,length(x),replace=TRUE)) #randomly sample with
replacement
      m_list[[iter]]<-sXX
      }
    }
  boot_clusters<-do.call("rbind",m_list) #combine the balanced matrices for each of the six types
into a single balanced matrix
  return(boot_clusters)
  }

#### FUNCTION TO OBTAIN BOOTSTRAP SAMPLE ####
get_boot_sample<-function (df,boot_iteration,boot_id_matrix) {
  df_list<-split(df[,c("pid","sid")],df[,"sid"])
  boot_list<-df_list[boot_id_matrix[,boot_iteration]]
  boot_ids<-do.call("rbind",boot_list)
  boot_ids_vector<-boot_ids$pid
  boot_sample<-df[match(boot_ids_vector,df$pid),]
  return(boot_sample)
  }

##### FUNCTION TO CARRY OUT BOOTSTRAP ANALYSIS #####
bootstrap<-function(df,fxn,outcome,n_boot_samples,boot_type="sampled"){
  point_estimate<-fxn(df,outcome)
  boot_id_matrix<-make_boot_id_matrix(df,n_boot_samples,boot_type=boot_type)
  result_list<-list()
  for (ith_sample in 1:n_boot_samples) {
    db<-get_boot_sample(df,ith_sample,boot_id_matrix)
    result<-fxn(db,outcome)
    result_list[[ith_sample]]<-result
    }
  result_matrix<-do.call("rbind",result_list)
  confint_lower<-apply(result_matrix,2,quantile,probs=.025)
  confint_upper<-apply(result_matrix,2,quantile,probs=.975)
  std_error<-apply(result_matrix,2,sd)
  if (ncol(result_matrix)>1) {
    confint_lower[6]<-exp(quantile(log(result_matrix[,6]),probs=.025))
    confint_upper[6]<-exp(quantile(log(result_matrix[,6]),probs=.975))
    std_error[6]<-exp(sd(log(result_matrix[,6])))
    }
  fmt_point_estimate<-point_estimate #round(point_estimate,round_digit)
  fmt_confint_lower <-confint_lower #round(confint_lower,round_digit)
  fmt_confint_upper <-confint_upper #round(confint_upper,round_digit)
  fmt_std_error<-std_error #round(std_error,round_digit)
  output<-
data.frame(t(t(names(point_estimate))),t(t(fmt_point_estimate)),t(t(fmt_confint_lower)),t(t(fmt_c
onfint_upper)),t(t(fmt_std_error)))
  colnames(output)<-c("coefficient","est","l95","u95","se")
  return(output)
  }

##### EXAMPLE FUNCTION CALLS #####
wgt_sate(mydata,"Y")
gcm_sate(mydata,"Y")
wgt_site(mydata,"D")
gcm_site(mydata,"D")

bootstrap(mydata,wgt_sate,"Y",1000,"balanced")
bootstrap(mydata,gcm_sate,"Y",1000,"balanced")
bootstrap(mydata,wgt_site,"D",1000,"balanced")
bootstrap(mydata,gcm_site,"D",1000,"balanced")
```
